# Lysozyme and bilirubin bind to ACE and regulate its conformation and shedding

**DOI:** 10.1038/srep34913

**Published:** 2016-10-13

**Authors:** Sergei M. Danilov, Heinrich Lünsdorf, Henry T. Akinbi, Andrew B. Nesterovitch, Yuliya Epshtein, Eleftheria Letsiou, Olga V. Kryukova, Tobias Piegeler, Elena Z. Golukhova, David E. Schwartz, Randal O. Dull, Richard D. Minshall, Olga A. Kost, Joe G. N. Garcia

**Affiliations:** 1 Department of Anesthesiology, University of Illinois at Chicago, Chicago, IL, USA; 2 Institute for Personalized Respiratory Medicine, University of Illinois at Chicago, Chicago, IL, USA; 3 Central Facility of Microscopy, Helmholtz-Center of Infection Research, Braunschweig, Germany; 4 Divisions of Pulmonary Biology and Neonatology, Cincinnati Children’s Hospital Medical Center, Cincinnati, OH, USA; 5 Department of Dermatology, Rush University, Chicago, IL, USA; 6 Faculty of Chemistry, Lomonosov Moscow State University, Moscow, Russia; 7 Institute of Anesthesiology, University Hospital Zurich, Zurich, Switzerland; 8 Bakulev Center for Cardiovascular Surgery, Moscow, Russia; 9 Department of Pharmacology, University of Illinois at Chicago, Chicago, IL, USA; 10 University of Arizona Health Sciences, Tucson, AZ, USA.

## Abstract

Angiotensin I-converting enzyme (ACE) hydrolyzes numerous peptides and is a critical participant in blood pressure regulation and vascular remodeling. Elevated tissue ACE levels are associated with increased risk for cardiovascular and respiratory disorders. Blood ACE concentrations are determined by proteolytic cleavage of ACE from the endothelial cell surface, a process that remains incompletely understood. In this study, we identified a novel *ACE* gene mutation (Arg532Trp substitution in the N domain of somatic ACE) that increases blood ACE activity 7-fold and interrogated the mechanism by which this mutation significantly increases blood ACE levels. We hypothesized that this ACE mutation disrupts the binding site for blood components which may stabilize ACE conformation and diminish ACE shedding. We identified the ACE-binding protein in the blood as lysozyme and also a Low Molecular Weight (LMW) ACE effector, bilirubin, which act in concert to regulate ACE conformation and thereby influence ACE shedding. These results provide mechanistic insight into the elevated blood level of ACE observed in patients on ACE inhibitor therapy and elevated blood lysozyme and ACE levels in sarcoidosis patients.

The extracellular domains of diverse membrane-anchored proteins, such as tumor necrosis factor α receptor (TNFR-α), L-selectin, ACE are released from the cell surface as soluble proteins through a regulated proteolytic mechanism - ectodomain shedding. Cell surface proteases such as the ADAMs (A Disintegrin And Metalloproteinase), as well as a variety of molecular intra-and extracellular interactions, regulate this process^1^.

Angiotensin-converting enzyme (ACE, CD143, EC 3.4.15.1), a Zn^2+^ carboxydipeptidase with two catalytic centers^2^, is a critical regulator of blood pressure and vascular remodeling^3^,^4^. Somatic ACE is expressed on the surface of endothelial and specific epithelial cells, as well as macrophages and dendritic cells^3^,^4^,^5^. Apart from membrane-bound ACE, blood and other biological fluids contain a variable amount of soluble ACE. Blood ACE originates primarily from the vast pulmonary microvasculature that exhibits 100% ACE expression compared to 10–15% ACE-positive capillaries in the systemic circulation^6^. ACE enters the circulating pool via shedding from the endothelial cell surface by an as yet unidentified ACE secretase^7^. In healthy individuals, the concentration of ACE in the blood is stable^8^ whereas significantly increased blood ACE is observed in subjects with sarcoidosis or Gaucher disease, consequently serving as a clinical biomarker of disease severity^9^.

We identified several ACE gene mutations that increase blood ACE levels (5–14 fold) including a mutation in the stalk region leading to greater ACE cleavage efficiency from the cell surface^10^, mutations eliminating expression of the transmembrane anchor and, therefore, resulting in direct ACE secretion into the circulation^11^,^12^, and a mutation residing at the interface of the N domain dimers (Y465D), affecting ACE dimerization and likelyincreasing accessibility of the stalk region to the ACE secretase^13^.

In this study, we identified a novel *ACE* gene mutation (Arg532Trp) that increases blood ACE activity (7-fold) and interrogated the mechanism by which this mutation significantly increases blood ACE levels. We proposed a novel regulation of ACE conformation, and as a consequence, ACE shedding via direct binding of circulating blood components - lysozyme and bilirubin to ACE.

Prior reports included several intracellular ACE-binding proteins - GRP78 (BiP), ribophorin 1, specific protein kinase C isoforms^14^, calmodulin^15^, ß-actin, non-muscle myosin heavy chain IIA^16^, integrins B1 and A5^17^, as well as an unidentified ACE-binding protein (14 kDa) in human serum^18^.

We now report the identification of lysozyme and bilirubin as ACE-binding blood components that act in concert to regulate ACE conformation and likely influence on ACE shedding. These results convey several biological and therapeutic ramifications including a potential explanation for elevated blood ACE level in patients on ACE inhibitor therapy.

## Results and Discussion

### Novel ACE mutation associated with elevated blood ACE activity

**Figure 1:**
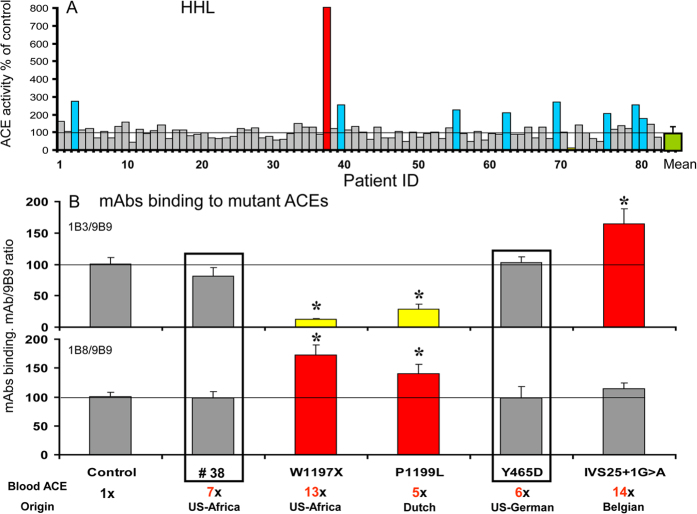
ACE activity and conformation in plasma samples. (**A**) ACE activity in 84 heparinized plasma samples from patients with a variety of pulmonary diseases (Hip-His-Leu as ACE substrate). Data was expressed as the % of individual ACE activity from mean value (green) for the healthy control group. Blue bars -samples with ACE activity higher than mean + 2SD. Red bar - the sample (#38) with putative ACE mutation. (**B**) ACE activity, precipitated by mAbs 1B3 and 1B8 from plasma of patient #38 and from plasma of subjects with different ACE mutations identified previously (W1197X^11^, P1199L^10^, Y465D^13^ and IVS25 + 1G > A^12^), was normalized to that precipitated with mAb 9B9^19^. Lower line in the legend to B – country origin of each mutation. Yellow bars-samples with mAb/9B9 ratio lower than 80% of control. Red bars-samples with mAb/9B9 ratio higher than 20% of control *p < 0.05 in comparison with mean value for control group.

Screening for ACE activity in plasma from 84 patients with sarcoidosis resulted in the identification of a case (#38) with markedly increased ACE activity (7-fold vs. control) (Fig. 1A). We explored potential mutations in the stalk region of ACE causing enhancement of its shedding^19^. Immunoprecipitation of ACE activity from the #38 plasma utilizing monoclonal antibodies (mAbs) directed to the stalk region failed to implicate the known stalk region mutations, P1199L^10^,^19^ or W1197X^11^, as both 1B3/9B9 and 1B8/9B9 binding ratios were similar to patients with normal ACE levels (Fig. 1B). We characterized the plasma ACE conformation from subject #38 using a panel of mAbs to 16 different epitopes of human ACE to generate a “conformational fingerprint of ACE”^20^. The immunoprecipitation profile of plasma ACE from subject #38 was similar, but not identical, to the “fingerprint” of plasma ACE from patient with the Y465D mutation outside the stalk region (Fig. S1A) that hinders ACE dimerization on the cell surface thereby increasing ACE shedding^13^.

We next sequenced all *ACE* exons and identified the R532W substitution in the N domain of the mature ACE protein (Fig. S1B). This residue is distant from the stalk region and is positioned on the other side of the N domain globule from the putative N domain dimer interface^13^. Subject #38 is heterozygous for R532W mutation (Fig. S1B) listed in GenBank as rs4314 (http://www.ncbi.nlm.nih.gov/projects/SNP/) and found in 5 out of 6220 chromosomes tested.

Western blot analysis on CHO cells expressing wild-type (WT) or mutant R532W ACEs demonstrated that this mutation failed to either alter ACE expression or impact ACE dimerization. We concluded that high plasma ACE activity in subject #38, carrier of ACE R532W mutation, is likely the result of changes in ACE shedding, possibly via conformational changes. This possibility was confirmed by the conformational fingerprinting of mutant R532W ACE, which revealed multiple conformational changes in the N and C domains and a striking decrease in the binding of mAb 6A12 (Fig. S2), which interacts with N domain residue 532.

### ACE-binding blood components regulate ACE shedding

What is the mechanism of this novel mutation in the N domain of ACE, very far from the stalk region, where membrane-bound ACE is cleaved by a still unidentified ACE secretase? How could this mutation change the rate of ACE shedding of the R532W mutant? We have demonstrated previously, that the binding of two mAbs to different, but overlapping epitopes on the N domain of ACE, 9B9 and 3A5, far from the interface of the N-domain dimer, including Y465 residue, results in 2–4 fold increase in somatic ACE shedding^21^. Therefore, the conformational changes in the N domain of ACE, induced by binding of some mAbs, could lead to conformational changes in the stalk region of somatic ACE and thus enhance shedding of somatic, two-domain ACE^21^,^22^.

**Figure 2:**
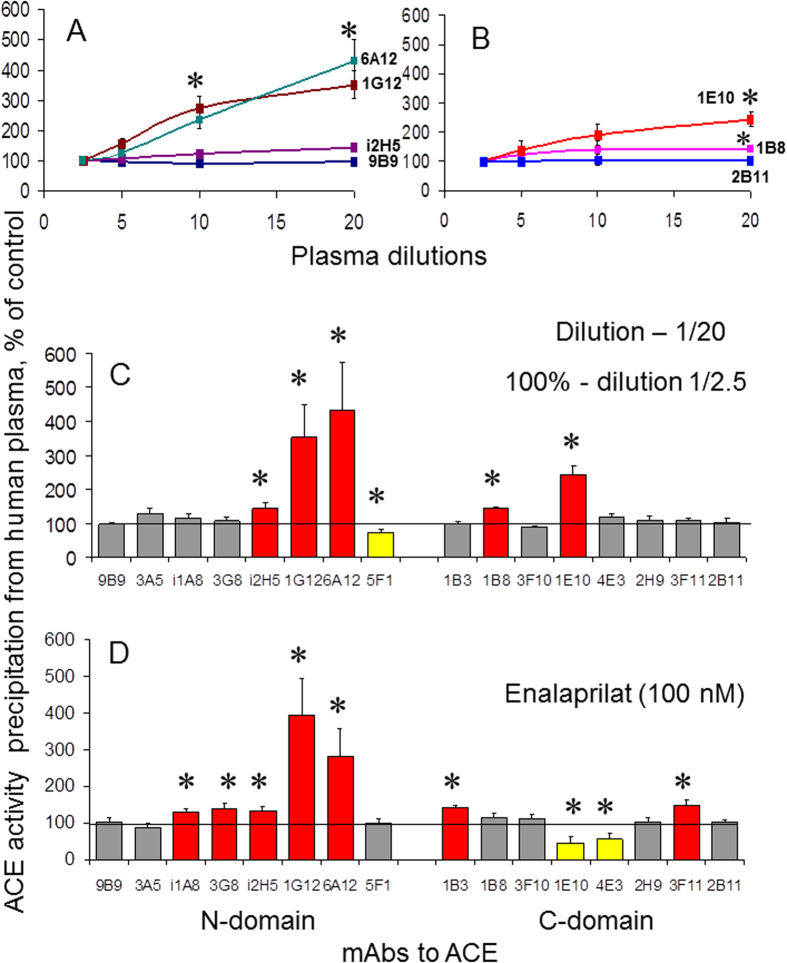
Effect of plasma dilution on mAbs binding to blood ACE. (**A**–**C**) Precipitation of ACE activity from plasma samples was performed with 16 mAbs to different epitopes of human ACE as in Fig. 1B using pooled human serum (from 10 healthy individuals) at different dilutions, 1/2.5, 1/5, 1/10, and 1/20. (**A**,**B**) Data are expressed as a percentage of the ratio of ACE activity precipitation by chosen mAb at a given dilution to that of 1/2.5 dilution (100%). (**C**) Effect of 8-fold difference in dilution on ACE precipitation by all 16 mAbs (1/2.5 dilution −100%). (**D**) Effect of ACE inhibitor enalaprilat (100 nM) on ACE precipitation by this set of mAbs. Data are mean + SD of triplicates. Bars highlighted with orange and red - the values of precipitated ACE activity from mutant ACE were more than 20% and 100% higher, respectively, than these values for WT. Yellow bars-samples with mAb/9B9 ratio lower than 80% of control. Colored bars indicate that the differences in ratios are statistically significantly different (p < 0.05).

Dilution of human plasma/serum (as well as adding ACE inhibitor) led to the increase in the relative binding of several mAbs to epitopes on both N and C domains of ACE (Fig. 2A–C), whereas the binding of these mAbs to recombinant ACE or purified ACE from seminal fluid was not changed upon dilution (not shown). We hypothesized that compound(s) in the blood can bind to ACE, and, then, dissociate during dilution. They could not be endogenous ACE inhibitors, because the most significant increase in binding upon dilution (which induce dissociation of ACE inhibitors) was demonstrated for mAbs 1G12 and 6A12 (Fig. 2), whereas the binding of common ACE inhibitors to plasma ACE, just an opposite, dramatically increased the binding of these mAbs, (Fig. 2D). Therefore, the binding of mAbs 1G12 and 6A12 should be decreased upon dilution and dissociation of an inhibitor from the complex with ACE. Effects of ACE substrates on mAbs 1G12/ 6A12 binding to blood ACE were more modest: AI and BK (+100%) and substance P (+50%) (p < 0.05, not shown), while the effect of enalaprilat was rather huge - +300% (Fig. 2D).

**Figure 3:**
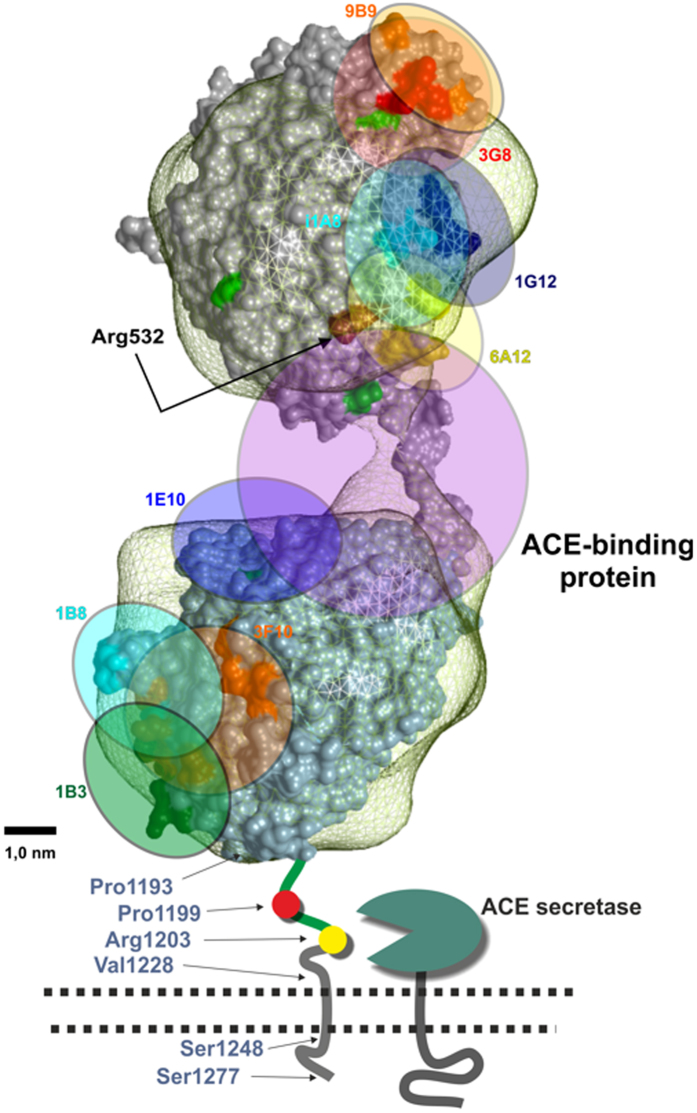
Putative complex of ACE with ACE-binding protein from the blood. A model of human somatic ACE was constructed using the model of porcine somatic ACE based on electron microscopy (EM)^59^. Specifically, the EM model was superimposed on the N domain of human ACE (PDB 3NXQ). The human C domain (PDB 1O86) was then superimposed on the C domain of the EM model. The epitopes were marked on the N and C domains as described previously^22^,^44^,^51^,^60^,^61^,^62^,^63^. Epitope mapping on the N and C domains was rendered in Chimera and PYMOL. The position of R532W substitution was shown by arrow. Putative ACE-binding protein was docked to the cleft between N and C domains of the model of the two domain human ACE in a way that to mask epitopes for mAbs 1G12/6A12 on the N domain and epitope for mAb 1E10 on the C-domain.

The positions of epitopes, whose mAbs binding to ACE increased upon dilution of the human plasma, also gave some additional information. Highly overlapping epitopes of mAbs 1G12 and 6A12 on the N domain are located close to the epitope for mAb 1E10 on the adjacent C domain (Fig. 3). Therefore, it is possible that an unknown compound(s) from the blood may bind to ACE covering regions on both ACE domains (Fig. 3), and subsequently dissociate during dilution.

ACE shedding from CHO-ACE cells was inhibited 2–3-fold in the presence of human and bovine serum (Fig. S3). R532W ACE shedding was comparable with that of wild-type ACE into the serum-free medium, whereas in the presence of 10% of human serum the rate of shedding of mutant ACE was ~2.5-fold higher than WT ACE (Fig. S3A), that is, the decrease of ACE shedding caused by the presence of serum was less pronounced for mutant ACE. We hypothesized that plasma/serum compounds, such as ACE-binding protein(s) and/or LMW components, stabilize ACE conformation on the cell surface thus preventing excessive shedding, while ACE mutant R532W exhibits diminished ability to bind these compounds from the serum and, therefore, is shed more effectively.

The effect of serum on the cleavage/secretion process of proteins from endothelial cells was studied in detail in the past. The total amount of proteins cleaved from their surface, especially higher MW proteins, dramatically increased in the presence of serum, likely due to protection of newly released proteins from degradation^23^. Serum was also shown to stimulate a cleavage of some membrane proteins, i.e. TNF-a precursor^24^. The decreased ACE shedding in the presence of serum indicates on a specificity of this effect, which can be directly connected to the high level of ACE in the blood of patient with R532W mutation. The rate of mutant ACE shedding in a serum-free medium was the same as for the wild-type ACE. In the presence of 10% of serum, however, it was significantly higher than that for WT ACE in the same conditions (Fig. S4A), that is, the decrease of ACE shedding caused by the presence of serum was less pronounced for mutant ACE.

We also compared the effects of compound BB-94 (batimastat), as well as mAbs 9B9 and 3A5 to ACE^21^, on WT and mutant ACE shedding from CHO cells. Impact of these compounds on ACE shedding in serum-free medium was identical for WT and mutant ACE, that is, similar increase of shedding. In the presence of 10% heat-inactivated human serum, however, the effect of these compounds on ACE shedding was less pronounced (about two-fold) for mutant ACE compared to WT ACE (p < 0.05, not shown).

The hypothesis about serum components, e.g. ACE-binding protein(s), which are able to stabilize ACE conformation thus preventing an excessive ACE shedding, likely allows us to correctly explain the well-known effect of ACE inhibitor treatment, namely, significant increase in ACE levels in the blood of patients^25^ and rats^26^,^27^. The enhancement of ACE shedding from CHO-ACE cells and from endothelial cells by ACE inhibitor enalaprilat was observed only in the presence of serum (Fig. S4B), which is in accordance with previous studies on isolated human endothelial cells^28^. We suggest that the binding of ACE inhibitors to ACE on the surface of endothelial cells (or on CHO-ACE cells) grown in the presence of 10% serum induces dissociation of plasma component(s)/protein(s) from the complex with ACE, which is reflected by the significant increase in mAbs 1G12 and 6A12 binding (Fig. 2D). The increased accessibility of the epitopes for these mAbs indicates that these epitopes could be putative binding sites for this hypothetical compound(s). In a consequence of such dissociation, ACE conformation could be destabilized and the rate of ACE shedding increased.

This effect of enalaprilat (and substance P, as an example of ACE substrate) on ACE expression and ACE shedding was further tested on several ACE expressing cells under serum-free growth conditions: artificial ACE-expressing cells, i.e. CHO, HEK and Rat Lung Microvascular Endothelial Cells (RLMVEC) transfected with recombinant human ACE, and natural ACE-expressing cells, HUVEC^29^. Among these cells, only HUVEC showed a modest increase in surface ACE expression by ~30% and a significant increase in the rate of ACE shedding by ~150% after 24 hours of incubation with enalaprilat (100 nM) in serum-free medium (p < 0.05, not shown) which is in accordance with previous studies on isolated human endothelial cells^28^. We therefore consider this fact as an indication that only HUVEC (from tested cells) could express this putative ACE-binding compound/protein, analogous to what, as we hypothesize, is present in human plasma.

Therefore, the binding of inhibitors to ACE results in conformational changes in the ACE molecule (reflected by the altered conformational fingerprint (Fig. 2D). This may induce dissociation of plasma component/protein from the ACE complex. As a consequence, ACE conformation on the cell membrane could be destabilized, thereby increasing accessibility of ACE to secretase.

Another argument to support the hypothesis about stabilizing effect of blood component (-s) on ACE conformation and shedding is the rate of shedding of the shorter, one-domain testicular ACE (tACE). This rate was reported to be several times higher in comparison with the full-size, two domain somatic ACE (sACE), which was expressed under identical conditions in CHO cells^30^,^31^. The authors proposed that the N-terminal domain of sACE acts as a “conformational inhibitor” that sterically hinders access to the stalk region for secretase^30^ or that the C-terminal domain contains a recognition motif for ACE secretase that is obscured by the N domain in somatic ACE^31^.

Our data allows us to hypothesize an additional explanation of a much higher rate of shedding of tACE. As shedding of both ACEs was estimated in the presence of 10% FBS, this difference could be due to less tight binding of the hypothetical ACE-binding serum component (ACE-binding protein) to the one-domain tACE isoform compared to the two-domain sACE, which, in turn, could lead to a higher rate of shedding of tACE. Therefore, we tested the effect of sera on the shedding of both tACE and sACEs from the surface of CHO cells - the shedding of full-size, two-domain ACE was dramatically inhibited by the presence of 10% serum, 34.5 + 6.5% from that in serum free-medium, which was equal to 20.2%/per24 hours (Fig. S3). The effect of human and bovine sera (10%) on the shedding of tACE (which, indeed, had much higher rate of shedding in serum-free conditions, 45.3%/per 24 hours) was much less pronounced, this rate being equal to 38.4 + 1.3%/per 24 hours in the presence of sera, that is, it was diminished only by 15.3 + 2.6% from the initial value (p < 0.05 for the effect of sera on sACE versus that on tACE). Therefore, significantly higher rate of testicular ACE shedding could also be explained by less effective binding of putative ACE-binding component/protein from serum to one-domain testicular ACE.

### Lysozyme binds to ACE

What proteins in the blood potentially bind to ACE on the endothelial cell surface? An unidentified ~14 kDa ACE-binding protein from human blood was previously reported^18^. Our analysis of human serum proteome data^32^ revealed several ACE-binding candidates including β2-microglobulin (13.6 kDa), lysozyme (14.5 kDa), cystatin C (13.4 kDa), transthyretin (13.7 kDa), and serum amyloid A protein (13.5 kDa). We tested β2-microglobulin and lysozyme by conventional techniques utilizing protein-coated microtiter plates followed by incubation with human plasma. Potential ACE binding was analyzed by precipitation of ACE activity on the immobilized proteins. We did not detect ACE binding by β2-microglobulin, while we registered modest lysozyme binding to ACE (Fig. S5A). We next assessed direct protein-protein interaction of purified lung ACE with these two plasma proteins by surface plasmon resonance (BIACORE). These studies validated that lysozyme, but not β2–microglobulin, binds to human ACE (Fig. S5B).

ACE conformation was altered by exogenous human lysozyme (Fig. S6), findings consist with direct ACE-lysozyme interaction. The epitopes for the select mAbs that alter binding in the presence of lysozyme are located on both N and C domains allowing us to conclude that lysozyme forms complexes with somatic ACE by occupying the cleft between domains. However, the majority of the epitopes for these mAbs are on the C domain including the epitope for mAb 1E10 that could be viewed as a part of the docking area for lysozyme.

ACE interaction with lysozyme, a well-studied protein, was unanticipated and had not previously been reported possibly reflecting weak binding constant of ACE-lysozyme interaction with very high association and dissociation rates of ACE-lysozyme complex (Fig. S5B) observed for ACE-caseinopeptide interactions^33^. Conversely, this may reflect the use of testicular ACE for co-immunoprecipitation in prior studies^14^,^15^,^17^, which does not have a cleft between domains. Moreover, low molecular weight proteins^14^ were unlikely to have been considered during co-precipitation experiments.

**Figure 4:**
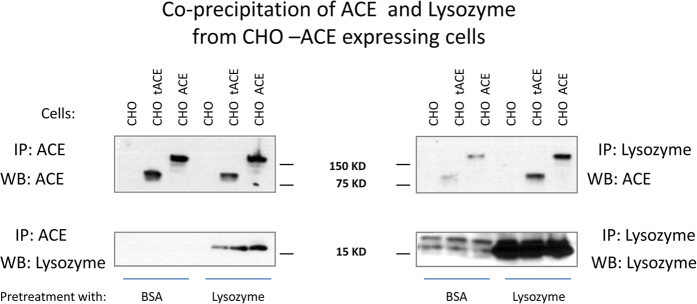
Identification of lysozyme as ACE-associated protein. CHO cells expressing testicular (CHO-tACE) and somatic (CHO-ACE) isoforms of ACE were pre-incubated overnight with human lysozyme or BSA (as negative control) at 250 μg/ml. Lysates of these cells were precipitated with anti-ACE and anti-lysozyme antibodies. Representative Western blots showed co-precipitation of ACE and lysozyme from CHO-ACE cells. Identical results were obtained in two additional experiments.

To further validate ACE-lysozyme interaction, we performed immunoprecipitation of CHO cells expressing testicular (tACE) or somatic (sACE) isoforms of human ACE with anti-ACE or anti-lysozyme antibodies. Lysozyme was co-precipitated with both ACEs by anti-ACE mAb, and both ACEs were co-precipitated with lysozyme by anti-lysozyme antibody (Fig. 4). Flow cytometry of CHO cells expressing both isoforms of ACE with anti-lysozyme mAbs (Fig. S7) showed that CHO-sACE cells pretreated with lysozyme expressed 30% higher amount of lysozyme on the cell surface than CHO-tACE cells (Fig. S7C). Thus, this implies two-domain ACE possesses a more favorable docking area for lysozyme than the single-domain isoform.

The ACE-lysozyme interaction potentially explains the parallel increase in blood ACE and lysozyme concentrations in patients with sarcoidosis^34^,^35^ and in patients on corticosteroid therapy^9^,^35^ We hypothesize that lysozyme, which is synthesized and secreted mainly by cells of myeloid origin^36^, binds to ACE, highly expressed on macrophage and dendritic cell within sarcoid granulomas^5^,^20^,^37^. Lysozyme may therefore appear on the cell membrane in a complex with ACE and subsequently shed into the circulation along with ACE.

### Lysozyme regulates ACE shedding *in vivo*

ACE phenotyping of mice with altered lysozyme expression showed that circulating ACE activity was decreased (3-fold) only in the sera of lysozyme M-deficient mice (Fig. S8) compared to sera from WT mice. Although this result appears to contradict the hypothesis that lysozyme stabilizes ACE and reduce ACE shedding, this paradox is addressed by the fact that expression of lysozyme P is dramatically increased in lysozyme M knock-out mice^38^, especially in the lung^39^. Therefore, it is logical to conclude that in mice lysozyme P (not lysozyme M) can bind to ACE, stabilize its conformation, and thus, reduce ACE shedding.

**Figure 5:**
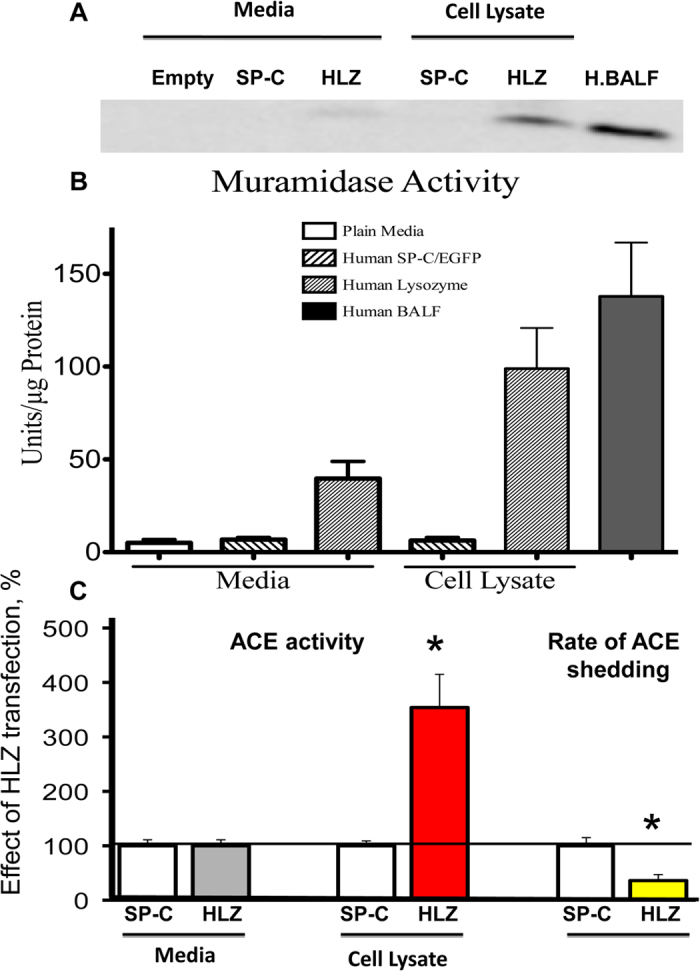
Effect of lysozyme expression on ACE shedding. CHO**-**ACE cells were transiently transfected with plasmids coding for human lysozyme (HLZ) or for surfactant protein C (SP-C as negative control). After 48 hours, ACE activity and lysozyme expression were quantified in the cell lysates and in the culture medium. (**A**) Western blotting of lysate and culture medium with anti-human lysozyme Ab. Positive control for the presence of lysozyme – human bronchoalveolar lavage fluid (H. BALF). (**B**) Lysozyme muramidase activity in the same samples was determined using killed *Micrococcus lysodeiticus*. (**C**) Soluble and cell-bound ACE activity was determined with Z-Phe-His-Leu as a substrate. The rate of ACE shedding was calculated as ACE activity in the culture medium/the total amount of cell-associated and secreted ACE activity. Data were expressed as a percentage from negative control. Mean + SD of three experiments.

CHO-ACE cells transiently transfected with plasmid encoding human lysozyme demonstrated decreased (3-fold) ACE shedding and reciprocally increased ACE content on the cell-surface (Fig. 5). This unambiguously confirms the direct effect of lysozyme on ACE shedding. This effect is similar to the effect of β2-microglobulin on the increase in surface expression of the T cell differentiation antigen^40^. Although speculative, ACE inhibitor-mediated ACE shedding from HUVEC observed even in the absence of serum (these study and refs 26,28) may reflect dissociation of lysozyme, endogenously expressed by endothelial cells^41^, from the ACE complex on the cell surface.

Previously, we measured blood ACE activity in different species^42^ and found that ACE levels may differ 10–20 fold. Data shown in Figs S9 and S10 allowed us to infer that differences in lysozyme amino acid sequences in various species could determine the ability of lysozymes to bind to ACE and influence on ACE shedding. This model (Fig. S10) does not exclude, however, the possibility for other blood proteins (from 14 kDa list) to occupy the cleft between domains in ACE globule and also participate in regulating ACE conformation and shedding.

### Identification of a LMW blood component that binds to ACE and regulates its shedding

We demonstrated that some low molecular weight (LMW) human plasma components also bind to human ACE, including endogenous ACE inhibitors (Fig. S11) as well as an unidentified LMW ACE effector which dissociates from blood ACE during filtration (Fig. S12), dialysis (Fig. S13), or dilution (Fig. 2), and changes the binding of mAbs 1G12 and 6A12 to the N domain of ACE (Figs S12 and S13). This LMW ACE effector is hydrophobic, weakly negative (Fig. S14), with a molecular weight less than 3 kDa (Fig. S13).

We tested the effects of human serum, depleted of this effector by filtration through a 3 kDa filter, on ACE shedding from CHO-ACE cells. While the 3 kDa serum filtrate alone did not affect ACE shedding (not shown), the rate of ACE shedding was 20–30% higher in the presence of heat-inactivated serum depleted of 3 kDa components compared to the whole pre-heated serum (Fig. S15). These data indicate that a specific LMW effector participates in the regulation of ACE shedding and the combined effect of lysozyme and this novel effector in the human serum on ACE shedding is more pronounced than with lysozyme alone. The effect of human plasma 3 kDa filtrate on ACE conformation is in accordance with this interpretation: this filtrate changed the conformation of both domains of ACE in human serum containing lysozyme (Fig. S16A), whereas it changed only the conformation of the N domain in purified ACE, mainly at the epitopes for mAbs 1G12 and 6A12 (Fig. S16B).

The effect of ACE inhibitor, enalaprilat, and EDTA on the conformation of ACE in the blood (containing both lysozyme and LMW ACE effector) and on the conformation of recombinant ACE, where both compounds are absent (Fig. S17), as well as the effect of chloride depletion (Fig. S18), suggest that dissociation of LMW ACE effector potentially depends on the nativity of Zn^++^ - N domain chloride binding site interaction^43^.

To identify the LMW ACE effector(s) present in human blood, the 3 kDa filtrate of human citrated plasma was analyzed by chromatography in combination with a mAb binding assay (Fig. S19), as well as mass spectrometry (Table S1). Fractions 33–34 increased precipitation of ACE activity by mAb 1G12 (Fig. S19) that can be attributed to the action of endogenous ACE inhibitory peptides^44^ (Table S1), whereas fractions 36–37, as opposite, decreased precipitation of ACE activity by this mAb. These fractions, therefore, contained unknown ACE effector.

**Figure 6:**
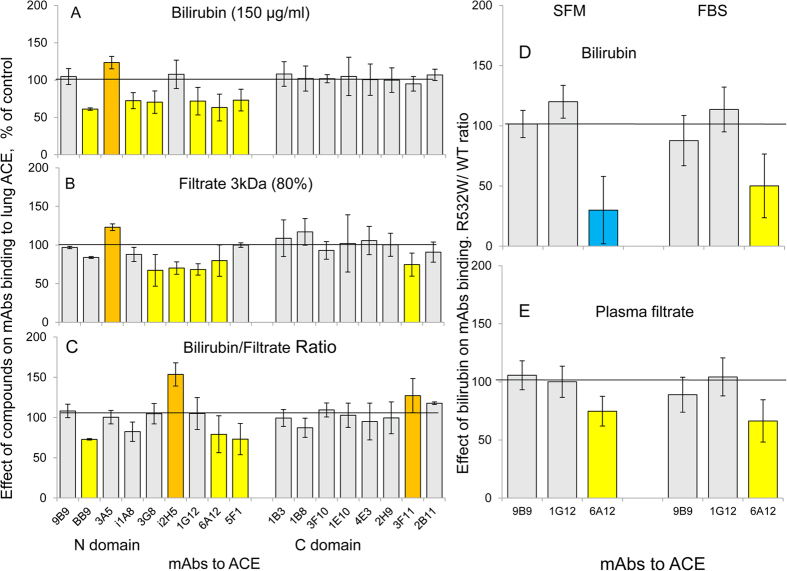
Effect of bilirubin and 3 kDa filtrate of human blood plasma on mAbs binding to pure human lung ACE, recombinant wild type ACE, and mutant, R532W. (**A–C**) Precipitation of ACE activity from samples was performed with 17 mAbs to different epitopes of human ACE as in Fig. 1B. (**A**) Lung ACE activity precipitation in the presence of 150 ug/ml bilirubin is expressed as a percentage from that without bilirubin. (**B**) Lung ACE activity precipitation in the presence of 80% 3 kDa filtrate of human plasma is expressed as a percentage from that without filtrate. (**C**) The ratio of the effects of bilirubin and 3 kDa filtrate to mAbs binding to ACE. Effect of 150 μg/ml bilirubin (**D**) and 3 kDa filtrate of human plasma (**E**) on ACE activity precipitation from soluble recombinant R532W mutant in comparison with wild-type ACE and is expressed as a percentage from the effect on WT ACE. Data are mean + SD of 3–8 experiments (depending of mAbs and types of experiments). Statistically significant (p < 0.05) increase in values of more than 20% of control was highlighted in orange, decrease in values of more than 20% - in yellow; decrease in values of more than 50% - in blue.

An analysis of the fractions by mass spectrometry did not allow definitive identification of the ACE effectors from human blood as there were excessive number of compounds (>60 in fractions 36–37, not shown). As an alternative approach, we performed bioinformatic analysis of the human metabolome, containing more than 7000 compounds^45^, and identified several candidates (Table S2), including bilirubin, a primary candidate due to the significant decrease in blood ACE levels observed in icteric patients, which inversely correlates with bilirubin levels in the blood^46^. Bilirubin alone decreased precipitation of lung ACE by several mAbs, including mAbs 1G12 and 6A12, similar to the effect of 3 kDa filtrate of human plasma (Fig. 6A–C). Thus, bilirubin potentially binds to ACE and regulates ACE conformation. Bilirubin alone (as well as the 3 kDa filtrate), however, did not affect the rate of ACE shedding from CHO-ACE cells (not shown). According to our estimation, ACE concentration in the lung is ~130 nM (and in the blood ~3 nM), while the total bilirubin concentration in the blood is about 200 μM (and free bilirubin is 10% of this amount ~20 μM) suggesting the possibility that the majority of ACE molecules are bound by bilirubin. This would imply a central role for bilirubin binding in regulation of ACE shedding.

Bilirubin, as well as 3 kDa filtrate of plasma, altered mAb 6A12 binding to ACE mutant, R532W, much stronger than to WT ACE (Fig. 6D,E). As the position of R532 is located within the epitope for mAb 6A12 (Fig. 3), we hypothesized that, while the mutation has already changed the conformation of ACE in the region of the epitope for this mAb, the binding of bilirubin further dramatically changed the local conformation of mutant ACE, thus preventing the binding of lysozyme to mutant ACE molecule, which, in turn, resulted in increased ACE shedding.

### Modeling of lysozyme-ACE interaction

We attributed the single R532W substitution in the N domain of ACE, leading to significantly increased ACE shedding, to gross conformational changes of somatic, two-domain ACE and the inability for ACE to bind blood components (such as bilirubin and lysozyme), that normally stabilize ACE conformation on the cell membrane.

**Figure 7:**
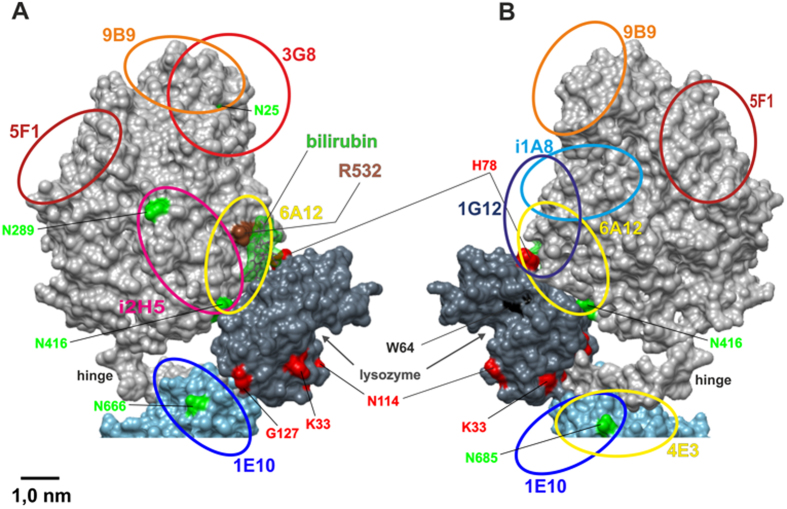
The model of lysozyme/bilirubin docking to somatic ACE. A model of human somatic ACE was constructed using model of porcine somatic ACE^59^ - projections (**A**,**B**) The epitopes for mAbs were marked on ACE with circles/ellipses, corresponding to the epitope size. Amino acid residues on the lysozyme structure (PDB 1W08): tested lysozyme mutation W64 - black spot; candidates for binding sites to ACE (from Fig. S9 – red). Bilirubin (green) covers the R532 within the epitope for mAb 6A12 and occupies the interspace between the N-domain and lysozyme H78.

Data on lysozyme sequence analysis (Fig. S9), effects of human serum and its components (lysozyme and LMW filtrate) on mAbs binding to blood and purified ACEs (Fig. 6, Figs S12–S19) led us to propose a model of lysozyme and bilirubin docking to ACE (Fig. 7). In this model, lysozyme primarily binds to the region of the epitope for mAb 1E10 on the C domain of ACE and occupies the cleft near the bridge between N and C domains touching the epitopes 1G12/6A12 on the N domain, while bilirubin (or bilirubin conjugates, mono- and di-glucoronides) binds only to the N domain of ACE, in particular, to the region of the epitope for mAb 6A12 containing R532W. Our data indicate that lysozyme, likely in combination with bilirubin, binds to ACE “stapling” the domains together and, thus, stabilizes ACE conformation on the surface of endothelial cells, which, in turn, mitigates ACE shedding into the circulation.

The mechanistic insights into ACE shedding elucidated may have clinical and biological significance. First, this may explain the long-observed increase in blood ACE protein in patients treated with ACE inhibitors^25^,^26^. We speculate that dissociation of lysozyme/bilirubin complex from ACE on the vascular surface enables a conformational change upon ACE inhibitor binding that subsequently leads to increased ACE shedding by an as of yet unidentified secretase.

The ACE-lysozyme/bilirubin hypothesis may explain the paradoxical simultaneous increase in substance P and ACE concentration in patients with migraine^47^: binding of substance P (ACE substrate) to ACE also induces dissociation of lysozyme/bilirubin from ACE, thus leading to higher rates of ACE shedding.

Our data may also explain the low level of ACE in urine^42^, despite high ACE expression in proximal tubule epithelial cells^5^. As lysozyme is freely filtered at the glomerulus, and reabsorption occurs almost exclusively in the proximal tubule^48^, the huge lysozyme concentration there could stabilize ACE on the surface of tubule epithelial cells thereby preventing ACE shedding into urine.

Finally, the ACE-lysozyme hypothesis may explain the parallel increase in blood lysozyme and ACE levels observed in patients with sarcoidosis, a systemic granulomatous disorder^9^,^34^,^35^. Lysozyme can be simply shed from the surface of dendritic cells into the blood as a complex with ACE - which concentration is exteremely high on these cells^20^,^37^.

In conclusion, we have identified a novel ACE mutation, R532W, which is associated with elevated blood ACE levels. Bilirubin and lysozyme were identified as novel ACE-binding blood components that act in concert to regulate ACE conformation and possibly influence ACE shedding. Together, these results provide novel mechanistic insights into the elevated blood ACE levels observed in patients on ACE inhibitor therapy with additional biologically and clinically relevant ramifications.

## Methods

### Study participants

The study was approved by the Institutional Review Boards of the University of Chicago and the University of Illinois at Chicago. All corresponding procedures were carried out in accordance with institutional guidelines. Eighty four patients were chosen for the TRIDOM study of the genetics of pulmonary diseases unrelated to this report. After providing written informed consent from all patients, citrated plasma was obtained from patients for determination of ACE activity and immunochemical characterization of ACE. One patient (#38) was found to have plasma ACE activity higher than 150 mU/ml (normal range 8–40 mU/ml^49^).

As an example of pure somatic ACE, we also purified ACE from seminal fluid according to^50^. Seminal fluid was obtained from ejaculates that were collected by Dr. V. Evdokimov (Institute of Urology, Moscow Russia) after obtaining informed consent from all donors and procedure was approved by IRB of this institution.

### ACE activity assay

ACE activity in serum/plasma, homogenates of mice lungs or culture fluids or lysates from ACE-expressing cells was measured using a fluorimetric assay with two ACE substrates, 2 mM Z-Phe-His-Leu or 5 mM Hip-His-Leu^49^. Briefly, 20–40 μl aliquots of samples were added to 200 μl of ACE substrate and incubated for the appropriate time at 37 °C. His-Leu product was quantified via complexing with *o*-phtaldialdehyde.

### Immunological characterization of the mutant ACE (Plate immunoprecipitation assay)

Ninety six-well plates (Corning, Corning, NY) were coated with anti-ACE mAbs via goat anti-mouse IgG (Pierce, Rockford, IL) bridge^51^ and incubated with tested ACEs. Then plate-bound ACE activity was measured with substrate for ACE (Z-Phe-His-Leu) directly in the wells^51^.

### Sequencing and genotyping

Genomic DNA was isolated from the whole blood. All 26 exons of ACE gene were sequenced in patient #38 using pairs of primers^52^. After the discovery of the mutation (R532W), genotyping of the 100 unrelated individuals for the search of the same mutation was performed by another PCR-based restriction fragment length polymorphism assessment. The 573 bp DNA fragment was amplified by PCR with primers Ex11_CP29-CP30_R532W_Fw (TCACACCCTCAATCCACTTCTC, intron 10) and Ex11_CP29-CP30_R532W_Rv (ATTTGTGTCGCCCCATGCCAG, intron 11) using genomic DNA.

The restriction endonuclease *AgeI* cuts this 573 bp PCR product from individual with native, wild-type ACE into two fragments of 347 and 226 bp. In the case of R532W substitution, the restriction site for this restrictase disappeared and *AgeI* did not cut this PCR product.

### Surface Plasmon Resonance (SPR)

Interaction of human ACE and human lysozyme was analyzed by SPR using optical biosensor Biacore 3000 (GE Healthcare, Piscataway, NJ) at 25 °C. Human ACE isolated from human lung using affinity chromatography on Lisinopril-Sepharose as in^50^ was immobilized on the dextran surface of CM5 biosensor chips by two approaches. In the 1^st^ approach, ACE was covalently coupled to the dextran surface of CM5 biosensor chips to a density of 4000–5000 response units (RU) through amine groups (according to protocol). In the 2^nd^ approach, mAb to ACE, clone 9B9^51^, was covalently coupled to the dextran surface of CM5 biosensor chips to a density of 4000–5000 response units (RU) through amine groups, and then human ACE (500 nM) was applied over immobilized mAb 9B9. Pure recombinant human lysozyme, expressed in rice (L1667, Sigma-Aldrich, St. Louis, MO) in running buffer (150 mM NaCl, 20 mM HEPES, pH 7.4, 0.005% (v/v) surfactant P20), was applied over immobilized ACE by injection in triplicate of various concentrations (5 to 115 μM) using a flow rate of 20 μl min^−1^. In control experiments, the configuration was an opposite: human lysozyme was immobilized on the biosensor and human lung ACE was applied over immobilized lysozyme (as described above). Data were prepared by the method of “double referencing” where parallel injections of analyte over a control dextran surface were performed as well as running buffer injections over both the immobilized ACE and control dextran surfaces. To confirm the specificity of ACE-lysozyme binding, the following purified proteins were used instead of lysozyme: human β2–microglobulin, B2M (Sigma-Aldrich, St. Louis, MO), and bovine serum albumin, BSA (Pierce, Rockford, IL).

### Co-precipitation of ACE and other proteins

CHO cells or CHO cells, stably transfected with testicular (CHO-tACE^53^) or somatic (CHO-ACE^54^) isoforms of human recombinant ACE, were incubated (at confluence) during 16 hours with Serum-Free Medium (SFM) in the absence or presence of 250 μg/ml of BSA or human recombinant lysozyme (both Sigma-Aldrich, St. Lous, MO). Cells were next washed with PBS, lysed in Nonidet lysis buffer, pH 7.5, containing 25 mM Tris-HCl, 150 mM NaCl, 0.5% Nonidet P40 (v/v) and protease inhibitor cocktail (EMD Millipore, Billerica, MA), left on ice for 30 min and centrifuged at 10 000g for 5 min. After pre-clearing with protein G Sepharose 4 Fast Flow (GE Healthcare, Life Sciences, Piscataway, New Jersey), proteins were immunoprecipitated from the whole cell lysates with either 1) anti-ACE mAb 2B11 (culture fluid, dilution 1/10), directed to an epitope on the C domain of ACE and thus recognizing both testicular and somatic ACE^55^; or 2) rabbit polyclonal antibodies to human lysozyme (Thermo Scientific, Rockford, IL, dilution 1/50). The immunoprecipitates after incubation with protein-G-agarose beads, centrifugation and washing (3X) with PBS-Tween (0.05%) were heated with SDS-PAGE sample buffer and separated on a gradient SDS-PAGE (4–20%), transferred onto Immobilon-P membranes, immunoblotted using primary mouse anti-ACE mAb 1D8^56^ or rabbit polyclonal anti-lysozyme antibodies and, then, secondary antibodies, anti-mouse-Horse Radish Peroxidase (HRP), and anti-rabbit-HRP, correspondingly, both at 1/2000 dilution (GE Healthcare, Little Chalfont Buckinghamshire, UK). Visualization of immunoreactive bands was achieved by enhanced chemiluminescence using ECL reagent (Amersham Biosciences, Pittsburgh, PA).

### Flow cytometry of CHO-ACE cells

CHO-tACE or CHO-sACE cells expressing recombinant isoforms of human ACE were incubated with tested mAbs at 4 °C for 60 min. The following mAbs were used: 1) mAb 9B9 (10 μg/mL) directed to an epitope on the N domain of ACE and thus recognizing somatic ACE only^51^; 2) mAbs 4E3 and 2B11 (1/3 dilution of culture fluid) directed to the epitopes on the C domain of ACE and thus recognizing both somatic and testicular ACE^55^; 3) mAb to human lysozyme (10 μg/mL), clone BGN/06/961 (Cat. #MA1-83313-Thermo Scientific Pierce, Rockford, IL). Ascitic fluid from mice injected with NS-1 mouse myeloma cells (Cat. #M-8273, Sigma-Aldrich, St. Louis, MO) was used as a source of non-immune mouse IgG as negative control. Cells incubated with the non-immune mouse IgG at the same concentration as the primary mAbs were processed in parallel to determine the background fluorescence. Then cells were washed with Hank’s Balanced Salt Solution (HBSS, Cellgro, Manassas, VA) containing 0.5% BSA and 2 mM EDTA. FITC-conjugated anti-mouse IgG (5 μg/mL, eBioscience, San Diego, CA) was then added at 4 °C for 30 min. Following incubation, cells were washed and resuspended in the HBSS buffer. Propidium iodide (PI) (eBioscience, San Diego, CA) at 1 μg/mL concentration was added before Flow CytoMetry (FCM) analysis to identify dead cells. Samples were processed by an LSR Fortessa (Becton-Dickinson, FranklinLakes, NJ) and data were analyzed with the Summit Software (Beckman Coulter, Inc., Fullerton, CA). Debris, clumps (based on scattering characteristics) and dead cells (based on inclusion of PI) were excluded from the analysis. The percentage of lysozyme- or ACE-positive cells was determined by calculation of the percentage of cells having fluorescence higher than fluorescence of cells incubated with non-immune mouse IgG after the subtraction of background fluorescence from each histogram.

### Mice with altered lysozyme expression

All *in vivo* mouse methods/experiments were approved by and performed in accordance with Cincinnati Children’s Hospital Medical Center IACUC Committee guidelines and regulations. Four groups of mice, lysozyme-overexpressing transgenic mice (lysozyme^tg^), lysozyme M-deficient mice (lysozyme M^−/−^), lysozyme M- and P-deficient mice (lysozyme MP^−/−^), and strain/age-matched wild-type (WT) mice were used. Lysozyme^tg^ mice (FVB/N strain) were generated by targeting expression of the rat lysozyme cDNA to the distal respiratory epithelium under the direction of the 3.7-kb human surfactant protein-C (SP-C) promoter^57^. Muramidase (lysozyme) activity was increased 16-fold in the Broncho-Alveolar Lavage Fluid (BALF) of transgenic mice relative to WT mice. Knock-out lysozyme M^−/−^ mice were generated in the C57BL/6 background by insertion of the gene encoding enhanced green fluorescent protein into the lys locus, as previously reported^58^, and were back-crossed 10 generations with FVB/N mice to facilitate comparison with lysozyme^tg^ mice. Lysozyme MP^−/−^ mice were generated in the 129 Sv-Ev/Swiss Black background and speed-bred five generations into the FVB/N background (Akinbi HT, unpublished). For each experiment, 5- to 6-week old mice were compared with WT FVB/N mice.

### Bioinformatic (*in silico*) analysis of this novel ACE mutation

A model of human sACE was constructed using the model of porcine sACE based on electron microscopy (EM)^59^. Arrangement of X-ray 3D densities of the N and C domains (PDB-IDs: 3NXQ_chain A and 1O86) into the porcine somatic lung ACE electron microscopy model was performed 1) by alignment of N-termini to north and C-termini to south, along the longitudinal axis of the ACE-model, 2) by setting the domains’ X-ray 3D densities to 2,3 nm resolution and fitting them into the ACE-model semi-automatically with the “Fit in map” module, and 3) by shifting and rotating both domains manually to re-align them to the models longitudinal axis and to allow the N-domain Glu590 residue to reside proximal to the C-domain’s N-terminus optimizing the fit to the N and C domain linking residues Val591 to Gly610 (“bridge region”). The epitopes for mAbs to ACE were marked on the N and C domains (Figs 3 and 7) according to^22^,^44^,^51^,^60^,^61^,^62^,^63^.

### Site-directed mutagenesis and *in vitro* analysis of the mutant ACEs

cDNAs encoding mutant ACE protein were created by GenScript (Piscataway, NJ) by mutation of the **G**CG codon for Arg at position 532 (somatic mature ACE numbering^2^) to codon TCG for Trp in expression vector based on pcDNA3.1 + /Hygro (Invitrogen Corp., Carlsbad, CA) and containing the full-length somatic ACE cDNA controlled by CMV early promoter^54^. Plasmid DNA was sequenced and clones with desired mutation were selected for each mutation.

Plasmids carrying the coding sequence for somatic Wild-Type (WT) ACE and above mentioned mutant were expressed in CHO and HEK cells using Plus Reagent (Invitrogen Corp., Carlsbad, CA) for transient transfection and generation of stable cell lines. Also for some experiments we used CHO cells transfected with 1) soluble somatic ACE, WTΔ^64^, 2) testicular isoform of human ACE^53^; 3) truncated N domain, D629^65^. Culture medium (Ultra-CHO medium, Cambrex Bio-Science, Walkersville, MD or serum-free DMEM, respectively) from these cells was used as a source of the soluble ACE (wild type and mutants) for biochemical and immunological characterization. Lysate of these cells was obtained with detergent Triton X-100 (0.5% in PBS) and used as a source of a mixture of the membrane and soluble forms of WT and mutant ACE. In some experiments, Human Umbilical Vein Endothelial Cells (HUVEC) expressing ACE at early passages^29^ were used.

### Western blot analysis of mutant ACEs

Samples for SDS electrophoresis were equilibrated to ACE activity of 200 mU/ml (Hip-His-Leu as a substrate) and were run using gradient (4–15%) Tris-HCl pre-cast SDS PAGE gels (Bio-Rad Laboratories, Hercules, CA). After electrophoretic transfer of proteins, the microporous PVDF-Plus membranes were incubated with mAbs to sequential epitopes on human ACE, suitable for detection of the denatured ACE, 3C5, 1D8, 5C8^60^,^65^.

### Co-transfection of CHO-ACE expressing cells with lysozyme cDNA

Plasmids carrying the coding sequence for human lysozyme (OriGene, Rockville, MD) were transiently transfected in CHO-ACE expressing cells^54^ using lipofectamine (Invitrogen Corp., Carlsbad, CA) according to manufacturer’s recommendations. Culture medium from these cells, containing 10% of heat-inactivated (65 °C, 30 min) Fetal Bovine Serum (FBS), was used as a source of the secreted (soluble) ACE for biochemical and immunological characterization. Lysate of these cells obtained with lysis buffer described above was used as a source of a mixture of membrane and soluble forms of ACE.

### Statistical analysis

The results were considered to be statistically significant when the level of probability was 0.05 or less based on a non-parametric Mann-Whitney U-test.

## Additional Information

**How to cite this article**: Danilov, S. M. *et al.* Lysozyme and bilirubin bind to ACE and regulate its conformation and shedding. *Sci. Rep.* **6**, 34913; doi: 10.1038/srep34913 (2016).

## Supplementary Material

Supplementary Information
