# Supplementary material for: Lysozyme and bilirubin bind to ACE and regulate its conformation and shedding
Source: Sci Rep. 2016 Oct 13;6:34913. doi: 10.1038/srep34913 (PMC5062130; doi:10.1038/srep34913)
Supplement: Supplementary Information [file srep34913-s1.pdf]

Supplementary Information for:

Lysozyme and bilirubin bind to ACE and regulate its conformation and shedding

Sergei M. Danilov[1,2 #], Heinrich Lünsdorf[3], Henry T. Akinbi[4], Andrew B. Nesterovitch[5], Yuliya Epshtein[2], Eleftheria Letsiou[2], Olga V. Kryukova[6], Tobias Piegeler[1,7], Elena Z. Golukhova[8], David E. Schwartz[1], Randal O. Dull[1], Richard D. Minshall[1,9], Olga A. Kost[6], and Joe G.N. Garcia[2,10]

[1] Department of Anesthesiology, University of Illinois at Chicago, IL
[2] Institute for Personalized Respiratory Medicine, University of Illinois at Chicago, Chicago, IL
[3] Central Facility of Microscopy, Helmholtz-Center of Infection Research, Braunschweig, Germany
[4] Divisions of Pulmonary Biology and Neonatology, Cincinnati Children's Hospital Medical Center, Cincinnati, OH
[5] Department of Dermatology, Rush University, Chicago, IL
[6] Faculty of Chemistry, Lomonosov Moscow State University, Moscow, Russia
[7] Institute of Anesthesiology, University Hospital Zurich, Zurich, Switzerland
[8] Bakulev Center for Cardiovascular Surgery, Moscow, Russia
[9] Department of Pharmacology, University of Illinois at Chicago, IL
[10] University of Arizona Health Sciences, Tucson, AZ

[#]Corresponding author: **Sergei M. Danilov**, MD, PhD
Department of Anesthesiology,
University of Illinois at Chicago, Chicago, IL 60612
Phone: (312) 413-7526, FAX: (312) 996-9680
E-mail: danilov@uic.edu

1) Characterization of the mutant ACE from patient # 38 (Figures S1-S2). The patient # 38 was a 33-y/o African-American male with a history of sickle-cell disease, pulmonary hypertension, acute chest syndrome and severe pulmonary sarcoidosis. To characterize the conformation of patient's blood ACE, we applied "conformational fingerprint of ACE", using a panel of mAbs to 16 different epitopes located on the N and C domains of human ACE[20]. The immunoprecipitation profile of plasma ACE from patient #38 (the pattern of ACE precipitation by 16 mAbs to ACE) was similar, but not identical, to profile of plasma ACE from patients with mutation, Y465D, in the N domain of ACE (fig.S1A), far from the stalk region, which also causes increased ACE shedding due to the changed extent of ACE dimerization, which leads to the changes of ACE conformation in the stalk region and to increased ACE shedding[21].

We also tested the inhibitory efficacy of mAbs to ACE, 3A5, i2H5[51,61], 1G12 and 6A12[44,51], which are anti-catalytic towards the N domain in somatic ACE, and mAbs 1E10 and 4E3, inhibiting the C domain[55]. Inhibitory effect of mAbs 1G12 and 6A12 having overlapping epitopes[44,51] was significant on blood ACE from control individuals, 76.6 $\pm$ 5.7 % of residual ACE activity, but was abolished for blood ACE from patient # 38, 91.8 $\pm$ 3.9 %, p=0.029. These data strongly indicate on the conformational changes within both domains of mutant ACE.

The conformational fingerprint of plasma ACE from patient #38 (Fig. 1B and fig. S1A) allowed us to suggest that this patient might carry an ACE mutation located in the newly discovered interface of the N-domain dimer or close to Y465. We therefore sequenced 9th, 10th, and 12th exons of the ACE gene, that code this interface[13,66], from patient #38 and did not find any mutation. We continued our sequencing efforts and, to our surprise, found mutation, Arg532Trp, in 11th exon of ACE (amino acid numbering according to[2]) (fig. S1B) at position 532 in the N domain of the mature ACE protein – far from the stalk region (Fig. 3) and on the other

side of the N domain globule from where interface of the putative N domain dimer was previously localized (Fig. 8 in [13]). Subject #38 is heterozygous for R532W mutation (fig. S1B) listed in GenBank as rs4314 (http://www.ncbi.nlm.nih.gov/projects/SNP/) and found in 5 out of 6220 chromosomes tested.

The mAb-based conformational fingerprint of ACE[20] allowed us to distinguish carriers of the novel R532W mutation, or Y465D substitution, from carriers of ACE mutations in the stalk region, all having elevated levels of blood ACE (Fig. 1B, fig. S1A), but did not allow to distinguish between the first two mutations. To facilitate detection of the novel mutation, we developed restriction analysis of a PCR-amplified fragment of genomic DNA spanning 573 bp including part of intron 10, exon 11 and part of intron 11. The restrictase *AgeI* will cut the 573 bp PCR product from an individual with WT ACE into two fragments - 347 bp and 226 bp. The C/T substitution (leading to R532W) eliminates restriction site in this PCR product, giving no fragments after restriction. Therefore, restriction analysis with *AgeI* can distinguish individuals with elevated blood levels of ACE with R532W substitution from patients with elevated levels of ACE due to Y465D mutations (Table in the legend to fig. S1).

Putative conformational changes in ACE induced by R532W mutation were further tested on CHO cells transfected with plasmid encoding for this mutant using conformational fingerprinting[20] of mutant ACE. Previously we generated over 40 mutations in the N and C domains of ACE and demonstrated that amino acid residue substitution usually changed the binding of those mAbs whose epitopes contained this amino acid residue and did not affect remote epitopes[19,22,44,60-63]. However, the mutation of the single amino acid residue, R532W, induced the changed binding of at least 5 mAbs, whose epitopes are located very far from 532

amino acid residue (fig. S2), that is R532W mutation induced multiple conformational changes of ACE molecule.

The binding of mAb 6A12 was completely abolished in the mutant ACE with transmembrane anchor (fig. S2A) while less changed in the soluble form (fig. S2B). We showed previously that the binding of this mAb was completely abolished when R532W substitution was performed in the truncated N domain of ACE (see Fig. 5 in[44]). Thus, the presence of the C domain within soluble two-domain ACE somehow compensates the effect of mutation on the conformation of the N domain. This explains why the binding of mAb 6A12 to soluble ACE from the blood of patient #38 (which, in addition, is a mixture of mutant and WT ACEs due to heterozygosity) did not show dramatic changes of mAb 6A12 (fig. S1A). Moreover, the changes of other mAbs binding were quite different for membrane and soluble forms of mutant ACE (fig. S2), confirming a prior observation that the conformations of soluble ACE and ACE with transmembrane anchor could be significantly different (Fig. 2 in[20]), also supported by significantly different kinetic characteristics, including domain specificity, of solubilized ACE and ACE attached to the cell surface[67].

We think that the decreased binding of mAb 6A12 to mutant ACE with transmembrane anchor could be a direct consequence of R532W substitution, because the epitopes for mAb 6A12 (as well as 1G12) contain amino acid residue in 532 position right in the middle of these epitope areas (Fig. 3), whereas the changes of binding of other mAbs to mutant ACE (fig. S2) could reflect gross changes in overall ACE conformation.

Western blot analysis was performed on the total cell lysate and the medium harvested from CHO cells expressing WT ACE and R532W mutant ACE in order to clarify whether this mutation could alter somatic ACE expression or induce an excessive dimerization (as has been

found for Y465D mutation in the N domain of ACE[13]). The cell-associated (lysate) WT and mutant ACE expression was resolved at approximately 170 kDa and was found to be similar (not shown). We also did not find any ACE dimers (oligomers) of R532W mutant (as opposite to dimers which were visible for Y465D mutant - see Fig. 2 in[13]). These results showed that 1) the mutation has no aberrant effect on somatic ACE expression, and 2) does not induce ACE dimerization. Therefore, the data confirmed our suggestion that the elevated plasma ACE level observed in the individual with the R532W mutation is more likely the result of the changes in the shedding mechanism, probably due to the conformational changes in ACE globule induced by this mutation, than the result of increased protein expression or increase in ACE dimerization.

2) Search for ACE-binding blood components that regulate ACE shedding (Figures S3-S4). We hypothesized that an unknown compound(s) from the blood can bind exactly to the inter-domain cleft and interfere with the binding sites for these mAbs (Fig. 3). Moreover, we can interpret the increase in 1B8 binding upon dilution as a consequence of the dissociation of this compound from the docking area for mAbs 1G12/6A12 and 1E10, this dissociation destabilizing ACE conformation and causing conformational changes in the stalk region close to the epitope for 1B8, which, in turn, increase the rate of proteolytic cleavage of ACE from the cell surface by ACE secretase and, therefore, increase blood ACE level. The effect of serum on ACE shedding from ACE expressing cells is in favor for such scenario.

We tested the impact of human and bovine serum on ACE shedding from the surface of CHO and HEK cells, which over-express recombinant human ACE, and HUVEC cells, which express human ACE naturally. The extent of the serum effect appeared to vary depending on the type of both serum and cells. However, the rate of ACE shedding in the presence of human and bovine

serum (both native and heat-inactivated) could be dramatically diminished - up to 2-times (fig. S3), except for HEK cells for which the effect of sera on ACE shedding was minimal (if any). It is worth noting that HEK-ACE expressing cells are characterized by a 3-fold lower rate of ACE shedding, 5.3%/24h compared to about 20%/24 h for HUVEC and CHO cells (fig. S3). The level of ACE in ACE-expressing HEK cells was similar, however, to that for HUVEC. The reason for the absence of the effect of serum on ACE shedding from HEK cells is unknown. It is possible, however, that these cells synthesize more lysozyme than other cell types and, thus, additional amounts of lysozyme in serum-containing media does not influence on ACE shedding.

3) Lysozyme binds to ACE (Figures S5-S6). ACE-binding protein (around 14 kDa) was previously detected in human blood, but has not been identified[18]. There are several candidates in the human serum proteome[32] with similar molecular masses: β2-microglobulin, lysozyme, cystatin C, transthyretin, serum amyloid A protein.

We tried conventional techniques to identify interactions with ACE of two of putative ACE-binding candidates, β2-microglobulin (MW 13.6 kDa) and lysozyme (MW 14.5 kDa). Measurement of ACE activity precipitated from serum to microtiter plates covered with proteins of interest did not show any ACE activity when β2-microglobulin was used, while some ACE activity was detected with lysozyme (fig. S5A). Modest binding of ACE with lysozyme could be attributed to rather low binding constant, to necessary washing steps during the analysis, and, most important, to the presence of high amount of free lysozyme as competitive protein in plasma.

Direct measurement of binding of pure human lung ACE with two possible 14 kDa proteins from human plasma was also performed with surface plasmon resonance, which allowed

excluding at least washing steps. Lysozyme, but not β2–microglobulin, was found to be able to bind to purified lung ACE, immobilized on the surface of the sensor indirectly, *via* mAb 9B9 to human ACE (fig. S5B). Lysozyme did not bind to mAb 9B9 on the chip surface, however, indicating that the interaction ACE-lysozyme is specific. We did not detect the binding of lysozyme to ACE, when ACE was immobilized on the surface of sensor directly, or, alternatively, when lysozyme was directly immobilized on the surface of the sensor and purified human lung ACE was injected (not shown). It was shown previously that ACE immobilized directly on the surface of microtiter plate exhibited altered kinetic properties and some epitopes characteristic for denatured ACE became available[65]. So, ACE conformation on the plate surface could not be considered absolutely native, and for the proper functioning of ACE molecule the direct contact with polystyrene surface should be avoided. Thus, ACE-lysozyme interaction revealed by BIACORE analysis confirms interactions of both reactants in their native conformational state, that is, when ACE was not in contact with a chip surface. We can state that ACE-lysozyme complex is characterized by high association and dissociation rates noted previously for interaction of ACE with inhibitory caseinopeptides[33]. We failed, however, to calculate the equilibrium dissociation constant, K_d, for the complex ACE-lysozyme, as the increasing concentrations of lysozyme led to the formation of ACE complexes with multiple lysozyme molecules.

We also tested an effect of human lysozyme on the conformation of ACE using conformational fingerprinting of the enzyme with mAbs. The binding of few mAbs to the two-domain ACE, as well as to truncated N and C domains, changed in the presence of human lysozyme (fig. S6). The epitopes for these mAbs are located on the surface of both domains of

ACE with majority on the C domain and include the epitope for mAb 1E10 which we assume to be a part of the docking area for lysozyme.

There are several reasonable explanations why the binding of lysozyme and ACE was not revealed before: 1) rather low binding constant of lysozyme-ACE interaction with very high association and dissociation rates of the complex (fig. S5), which could constrain the detection of such interaction using co-immunoprecipitation technique; 2) the fact that testicular, one domain ACE, that had no native cleft between N and C domain as a docking surface for putative ACE-binding protein from the blood, was used for co-immunoprecipitation[14,15,17]; 3) even when somatic ACE was precipitated by anti-ACE mAb, low molecular weight proteins were not considered in co-precipitation experiments[16].

Flow cytometry of CHO-tACE and CHO-sACE cells with anti-lysozyme mAbs was performed with cells cultivated overnight with 250 ug/ml of human lysozyme (or with BSA as a negative control) before an experiment. Such a treatment leads to an internalization of significant amount of lysozyme (or BSA) inside the cells[68]. The internalization of lysozyme in CHO cells was confirmed by the binding of anti-lysozyme antibodies (fig. S7B). It was shown that lysozyme is able to interact with both ACE isoforms (fig. S7C). However, CHO-sACE cells pretreated with lysozyme expressed significantly higher amount of lysozyme on the cell surface than CHO-tACE cells (fig. S7C), thus confirming that lysozyme binds to the two-domain ACE more tightly. Partly, however, some amount of lysozyme on the surface of CHO cells (fig. S7C) could be explained by electrostatic interaction of positively charged lysozyme with negatively charged surface of mammalian cells[69]. We showed that pretreatment of CHO-ACE cells with positively charged protamine sulfate significantly (53%), but not completely, decreased the binding of anti-lysozyme mAbs to the surface of these cells (fig. S7D). We also demonstrated

that the binding of anti-lysozyme mAbs to the surface of CHO-ACE cells (i.e., the amount of lysozyme on the cell surface) slightly decreased in the presence BSA (1 mg/ml) (fig. S7D). Nevertheless, significant amount of lysozyme on CHO-ACE cells surface even in the presence of protamine sulfate argues in favor of the specific interaction of lysozyme with ACE on the surface of ACE expressing cells.

It is worth noting, in addition, that ACE inhibitor-mediated ACE shedding discussed above was observed for HUVEC (but not for other cells) even in the absence of serum[26,28]. This observation can be explained by the presence of lysozyme, endogenously expressed by endothelial cells[41], which can dissociate from the ACE complex on the endothelial surface in the presence of ACE inhibitors.

Moreover, lysozyme-ACE interaction allows us to suggest that once lysozyme appeared on the cells surface in a complex with ACE (during physiological ACE trafficking to the cell surface), it can be shed from the surface of ACE-expressing cells altogether with ACE and become soluble, thus providing a reasonable explanation of parallel increase in blood ACE and lysozyme in patients with sarcoidosis, but not in controls.

4) Lysozyme regulates ACE shedding *in vivo* (**Figures S8-S10**). Putative physiological effect of lysozyme on ACE shedding was explored on mice with altered lysozyme expression. Mice, in contrast to human, which has one lysozyme gene, have 2 genes, for lysozymes M and P[70]. We tested ACE activity in the serum and lung tissues of lysozyme M-deficient mice[39] (Lys $M^{-/-}$), lysozyme M and P-deficient mice (Lys $MP^{-/-}$ [Akinbi HT, unpublished]) and mice overexpressing rat lysozyme (Lys^{+++} in the distal respiratory epithelium under control of human surfactant protein C (SP-C) promoter[57]).

ACE activity in serum, correlating with the rate of ACE shedding, was significantly decreased (3-fold) in lysozyme M-deficient mice (fig. S8A). This result seemed to contradict to our hypothesis that lysozyme stabilizes ACE and prevents excessive ACE shedding, but it is known that knocking-out of lysozyme M leads to dramatic over-expression of lysozymeP[57], especially in the lung[71], where lysozyme P becomes expressed not only in macrophages[71], but also likely in the endothelial cells of the lung which represent the main source of circulating ACE[5]. Therefore, it is logical to suggest that in mice lysozyme P (not lysozyme M) binds to ACE, stabilizes its conformation, and, thus, prevents an excessive shedding of ACE.

We have found earlier that mice, rat and swine have much higher levels of blood ACE levels - 10-20-fold higher than human, or dog, or sheep, etc.[42], which confirmed previous findings[72,73]. We explored the possibility that differences in lysozyme structure from different species could determine blood ACE level due to putative differences in interactions with ACE. The starting point was that ACE expression in endothelial cells of different species (especially in the lung) could determine the level of ACE in the blood of these species. Then, the ability (or inability) of lysozymes from these species to bind to ACE on the surface of endothelial cells can contribute to the ACE shedding efficacy. We performed an alignment of lysozyme amino acid sequences from different species marking the level of ACE activity in the blood (fig. S9). Surprisingly, lysozymes from species with high levels of blood ACE (mice, rats and pigs, highlighted by red) possessed a lot of residues (marked also by red and yellow) which differ from those in lysozymes from species with low ACE level in the blood (human, monkeys, dogs, etc.). These residues could be considered as putative defining residues for a lower ability of lysozymes to bind to ACE and, therefore, stabilize its conformation and prevent shedding. On the other hand, we can mark some residues within lysozyme amino acid sequence which could be responsible for higher

binding of lysozyme to ACE. Thus, Leu8, Asp18, Lys(Arg)32, Trp33, His78, Val93, Ala94, Asn114, Leu(Val)121, Ile(Val)125, and Gly127 are characteristic residues for the majority of lysozymes belonging to the species with lower level of ACE in the blood. It is important that while lysozyme has pK around 11 and ACE has pK 4.5-4.8, lysozyme-ACE interaction in neutral media is not obvious ion-ion interaction but represents some kind of specific protein-protein interaction provided by these amino acid residues, mostly hydrophobic by nature.

It is worth noting, however, that even a definite lysozyme can, probably, “nestle” within the cleft between ACE domains in different ways (fig. S10), e.g., just in the opening or deeply inside, depending on definite ACE structure. Moreover, we cannot exclude the possibility that other small proteins from the blood might bind to ACE as well, taking the position in the cleft between ACE domains.

5) LMW component from the blood also binds to ACE and regulates ACE conformation (Figures S11-S19). Besides exploring lysozyme as ACE-binding protein from the blood, we considered the possibility that other blood components (non-proteins) could also bind to ACE and regulate its functions. We showed that, if to measure ACE activity at increasing dilutions (decreasing concentrations) of human serum, then ACE activity in the undiluted serum appeared to be 20-50% less than ACE activity determined in 1/10 diluted human serum (fig. S11). We showed that this effect was not caused by fluorescence quenching neither by high substrate/product concentration nor by high protein concentration in the undiluted serum. This phenomenon was considered as an evidence of the presence of endogenous ACE inhibitors in the media[74]. Interestingly, an effect of serum dilution on measurable ACE activity with a substrate ZPHL was higher than with another substrate, HHL (fig. S11B), which led to significant increase

in ZPHL/HHL ratio (the ratio of the rates of the hydrolysis of these two substrates by ACE in definite conditions) upon dilution. ZPHL/HHL ratio rose from 0.86 for undiluted serum to 1.00 for dilution 1/5, and further to about 1.20, close to the value for this parameter of 1.25 for recombinant ACE, for serum diluted 1/20. Because HHL is cleaved faster (9-fold) by the C domain, than by the N-domain[75] and ZPHL is cleaved approximately equally by both domains[51] we suggested that these results indicate on the presence of endogenous ACE inhibitors directed more to the N domain active center[42].

As we already demonstrated (Fig. 2C) adding of exogenous ACE inhibitors (for example, 100 nM enalaprilat) to the human serum dramatically increased the binding of two mAbs, 6A12 and 1G12, directed to the overlapping epitopes on the N domain, to serum ACE, confirming previous studies[44,76]. At the same time, simple dilution of serum, which inevitably results in dissociation of any inhibitor from the complex with ACE with expectable decrease of the binding of these mAbs, led, on the contrary, to a significant increase of relative binding of the same two mAbs to the N domain, as well as to an increase of binding of one mAb, 1E10, to the C domain of ACE. The epitope for mAb 1E10 on the C domain was previously localized in close proximity to the N domain of the enzyme, namely, close to the epitopes for mAbs 6A12 and 1G12 (see also Fig. 2). Similarly, binding of these mAbs, 1G12 and 6A12, to the N domain of serum ACE increased after serum filtration through Amicon 5 kDa filter (fig. S12C) along with the increase of the binding of mAb 1E10 to the C domain. Alternatively, adding of heat inactivated human serum (30%) to ACE within the serum diluted 1/10 led to a significant decrease in 1G12 and 6A12 binding and also to the increased binding of mAb i1A8, directed to the epitope on the N domain (fig.S12A). Besides, human serum5 kDa filtrate containing LMW compounds also decreased the binding of mAbs 1G12 and 6A12 to serum ACE (fig. S12B). These results argued

against 14 kDa protein from human serum as ACE-binding protein that masks epitopes for mAbs 1G12 and 6A12 on the N domain of ACE and rather indicate in favor of ACE effector with molecular weight less than 5 kDa.

In order to estimate the size of this putative ACE effector more precisely we dialysed human serum using dialysis tubes with different pores, 10 kDa, 3.5 kDa and 1 kDa, and filtrated serum through filters with different kDa limits. Dialysis/filtration through pores with 3.5-10 kDa limit led to a dramatic increase in mAbs 1G12 and 6A12 binding to blood ACE, whereas depletion of human serum from compounds with molecular weight less than 1 kDa led, on the contrary, to a significant decrease in the binding of these very mAbs (fig. S13). We can interpret these results in a way that serum contains i) ACE effector(-s) with molecular weight less than 3.5 kDa which forms complex with ACE decreasing, thus far, mAbs 1G12 and 6A12 binding, while its dissociation during dialysis, filtration and even dilution results in the increase of these mAbs binding; and ii) endogenous ACE inhibitory peptides (less than 1 kDa) whose depletion decreases 1G12/6A12 binding which is consistent with our previous finding[44,76]. The binding of mAb 1E10 also increased as a result of a depletion of human serum from compounds with molecular weight less than 5 kDa, whereas the depletion from compounds with MW less than 3.5 kDa did not result in any change in this mAb binding. Altogether, these results may indicate on the presence of different ACE effectors in human serum.

In order to define a chemical nature of LMW ACE effector(-s) from human serum, we preliminarily precipitated serum HMW proteins by 2 volumes of acetonitrile (ANC), which is known to be able to precipitate up to 90% of serum proteins with molecular weight more than 20 kDa[77]. The resultant ACN supernatant of human serum (10%) significantly decreased mAbs 1G12/6A12 binding (fig. S14A) to ACE in diluted plasma, similar to the effect of 5 kDa filtrate

of human serum (fig. S12B). We passed this ACN supernatant through the reverse phase SEP-PAK column S18, as well as through ion exchange spin Vivapure columns (Sartorius, Edgewood, NY) - strong cation exchanger column S and weak anion exchanger column D. An ability of ACN supernatant to decrease mAbs 1G12/6A12 binding to blood ACE disappeared after passing through SEP-PAK column S18 and through anion exchanger (fig. S14B, C). Passing of ACN supernatant through cation exchanger did not change the ability of ACN supernatant to decrease mAbs 1G12/6A12 binding (not shown). Thus, we can conclude that LMW ACE effector is hydrophobic and, at the same time, anionic compound.

All these results allowed us to conclude that human serum contains at least several ACE effectors/ACE binding proteins/ACE inhibitory peptides:

1) Binding of ACE binding protein with MW likely 14 kDa masks the epitopes for mAbs 6A12 and 1G12 on the N domain and epitope for mAb 1E10 on the C domain of ACE;

2) Binding of ACE effector with MW less than 3.5 kDa influences mainly the epitope for mAb 1G12 and 6A12 on the N domain of ACE, decreasing these mAbs binding;

3) Binding of endogenous ACE inhibitory peptides with MW less than 1 kDa (or exogenous, commercially available ACE inhibitors) to human blood ACE increases mAb 6A12/1G2 binding to blood ACE likely via dissociation of ACE-binding protein and LMW ACE effector(-s). from mainly epitopes for mAb 1G12 and partially epitope for mAb 6A12 and via dissociation of ACE-binding protein with higher molecular weight (likely 14 kDa) from region covering epitopes of mAb 1G12 and 6A12 on the N domain and epitope for mAb 1E10 on the C domain.

In order to assess the possibility that this novel LMW ACE effector(-s) can influence on ACE shedding, we tested the effects of human serum, which was depleted of this effector by filtration through filters with pores of different size, on ACE shedding *in vitro*. CHO-ACE

expressing cells were cultured 24 hours in Serum-Free Medium (SFM) in the absence or presence of different additives. ACE shedding in the presence of human serum depleted of compounds with MW less than 3 kDa (i.e. without a putative ACE effector) was statistically higher than in the presence of the same concentration (10%) of human serum that was not filtered (fig. S15). Filtration of the same serum with added exogenous ACE inhibitor enalaprilat (which likely induces dissociation of putative protein/compounds from the complex with ACE in the blood) through filters with pores of different sizes showed different effects. Enalaprilat only slightly increased ACE shedding in the presence of human serum which was filtered through 3 kDa filter, but significantly increased shedding in the presence of serum which was filtered through 30 kDa filter (fig. S15A). As a positive control for this assay, 10% heat-inactivated human serum and human lysozyme (1 mg/ml) similarly (-25%) decreased ACE shedding. Solution of BSA at the same concentration (1 mg/ml) also decreased shedding, albeit to a lesser extent (-15%), but the depletion of the same preparation of BSA from relatively low molecular weight components (ACE effector and lysozyme) by passing through 30 kDa filter completely abolished its effect on ACE shedding, thus confirming that some compound(-s) with MW less than 30 kDa contaminating BSA preparation influence the enzyme shedding. These results coincide with the abovementioned data that lysozyme is the main regulator of ACE shedding, but also show that some LMW effectors could influence on ACE shedding as well.

Due to its strong positive charge at physiological pH (pI ~ 11) lysozyme forms complexes (heterodimers) with several macromolecules: α_2-macroglobulin, albumin, α-lactalbumin. Lysozyme also binds numerous small molecules present in the blood: inositol hexaphosphate - IP_6 or phytate[78], flavones and many drugs[79]. Consistent with possibility that lysozyme could bind to ACE in complex with some other proteins or small molecules we identified that lysozyme in

complex with inositol hexaphosphate more effectively inhibited ACE shedding from CHO-ACE expressing cells, than lysozyme alone (+ 11.0 % of inhibition compared to lysozyme, $p<0.05$).

We suggest that both ACE binding compounds from the blood, lysozyme and novel LMW ACE effector(-s), decreased ACE shedding after binding to ACE. However the combined effect of these compounds on ACE shedding is more pronounced. Effect of human plasma 3kDa filtrate on ACE conformation is in accordance with this interpretation: this filtrate changed the conformation of the surface of both domains of ACE in diluted plasma (fig. S16A), which genuinely contains lysozyme, while changing only the conformation of the N domain, mainly at the epitopes for mAbs 1G12 and 6A12, of purified ACE (fig. 16B).

The concert action of lysozyme and LMW effector was confirmed by the experiments with serum dilution, which demonstrated that the effect of dilution on the binding of 1G12/6A12 and 1E10 (Fig. 2) was less pronounced ($p<0.05$) if original serum was preliminarily depleted of LMW compounds by passing through "desalting" ZEBA column with 5 kDa limit ($p<0.05$, not shown). So, the biggest effect on mAbs binding to blood (serum) ACE could be attributed to a complex of ACE effector (MW less than 5 kDa) and ACE binding protein.

It is interesting that not only specific ACE inhibitors caused the increase of mAbs 1G12/6A12 binding to blood ACE, but EDTA affected similarly (fig. S17), albeit less pronounced (250-300% compared to 400-700% for enalaprilat). We cannot exclude the possibility that this effect in both cases (only slight for purified ACE) is due to dissociation of some compound(s) from the blood which was bound to ACE.

The effect of Cl^- depletion on mAbs binding to pure ACEs was significant not only for mAbs 1G12 and 6A12, but also for mAbs i1A8 and 3G8 (fig. S18), which epitopes[63] are close to the N domain chloride binding site[43]. It was shown recently that chloride-binding center can interact

with zinc-anion in the active center of ACE[43]. As EDTA is complexing Zn^{++} from ACE active centers we can suggest that putative dissociation of LMW ACE effector upon action of EDTA could be due to the disturbance of Zn^{++} - chloride binding site interaction within the N domain.

In order to define a chemical nature of LMW ACE effector from human blood, the 3 kDa filtrate of human plasma was analyzed further by gel-permeation chromatography (fig. S19). Fractions from these chromatography cycles were undergone to antibody binding assay, namely, they were tested for their effect on mAbs 1G12 and 9B9 binding efficacy to plasma ACE (fig. S19). It was demonstrated that different fractions had opposite effects on mAb 1G12 binding to ACE (there was no effect on mAb 9B9 binding), some fractions increasing this mAb binding, while other decreasing. The fractions which increased precipitation of ACE activity from human citrated plasma (diluted 1/5) by mAbs 1G12 (fractions 33-34 in fig. S19B) likely contain a mixture of ACE inhibitory peptide(-s) with MW more or around 1 kDa, whereas fractions (36-37 in fig. S19B) which decreased precipitation of ACE activity from human plasma (and activity of purified seminal fluid ACE, not shown) by mAbs 1G12, likely contain some ACE effector(-s) with molecular weight less than 1 kDa.

6) Modeling of lysozyme-ACE interaction (Figure 7). The alignment of amino acid sequences of lysozymes from different species in accordance with blood ACE activity in these species indicates that lysozyme may bind ACE in the positions 8, 15, 18, 27, 32-33, 78-80, 93, 93-94, 114, 121-122, 125-127 (fig. S9). According to our structural model of lysozyme/bilirubin docking to ACE (Fig.7), where these putative lysozyme binding sites to ACE are colored red, the lysozyme's C terminal residues appear to dock to the ACE C domain, especially to the region of the epitope for mAb 1E10 (marked in blue, Fig. 7 A-B), while lysozyme molecule occupies the

cleft between ACE domains. In that scenario those residues of lysozyme that are supposed to be in contact with the N-domain are D18, H78, V93, A94; those supposed to be in contact with the C-domain are V121, V125 and G127. Bilirubin, shown in transparent green, appears to occupy the inter-space between the region of the N-domain, including R532, and lysozyme H78, this region on the N domain being also a docking surface for mAbs 1G12/6A12.

It is worth noting that we measured serum ACE activity in 2 patients with W64R mutation of their lysozyme (versus their 4 relatives without mutation) and found no difference in ACE serum levels of carriers and non-carriers of this mutation (not shown). This lysozyme mutation, as well as 5 others located in the same region of the native structure of lysozyme, namely in the β-domain, have been found to be associated with systemic non-neuropatic amyloidosis (see for review[80]). Because W64R is located far from the predicted docking area of the lysozyme/ACE interface we can consider the absence of the effect of this mutation on serum ACE levels as a support of our model of lysozyme docking into ACE inter-domain cleft.

7) Biological and clinical implications of lysozyme/bilirubin binding to ACE.

Previously, the molecular mechanisms underlying the marked (5-fold) family increase of plasma ACE were discovered: Firstly, P1199L substitution led to enhanced accessibility of the stalk region for ACE secretase and for the increment in the cleavage-secretion process[10], while W1197Stop mutation led to the direct trafficking of ACE to the cell media[11]. Secondly, the Y465D substitution in the N domain of ACE (far from the stalk region) changed the extent of ACE dimerization[13]. Thus, the overall ACE conformation, including supposedly the stalk region, on the endothelial cell surface appears to be altered and, as a result, ACE shedding was increased. Thirdly, we identified a mechanism of elevation of blood ACE in patient #38. Here,

the R532W substitution likely prevents proper binding of ACE effectors/binding proteins from the blood (bilirubin and lysozyme) to the inter-domain cleft between N and C domains, which results in decreased stabilization of ACE on the cell surface, more flexibility of the enzyme molecule and, as a consequence of that, an increased ACE shedding and elevation of ACE level in the blood. The search for these mutations is of considerable clinical importance. Genetically determined elevation of the blood ACE level may lead to false diagnosis of sarcoidosis, which is often accompanied by elevated ACE levels, and consequently to unnecessary long-term immunosuppressive treatment[81]. Since blood ACE elevation is often taken as a marker of disease severity (sarcoidosis and Gaucher diseases), it is important for clinicians and medical scientists to be aware of alternative genetic causes of elevated blood ACE, not apparently linked to disease. Thus, whenever very high serum ACE is found in the evaluation of potential sarcoidosis, we recommend mutations in P1199L[10,19], W1197X[11], Y465D[13], INS25+1G>V[12] and the novel R532W reported here to be considered.

The facts that lysozyme and bilirubin both bind to ACE allows to explain several facts, which are important for clinical and biomedical scientists. One of them is a paradoxical simultaneous increase in ACE level and substance P level in the blood in patients with migraine[47]. Substance P is a natural substrate for ACE[82] and, therefore, an increase in blood ACE (found in patients with migraine) apparently could be accompanied by the decrease in substance P concentration. However, in reality the situation is quite an opposite. Binding of substance P to ACE induced a conformational change in the epitopes for mAbs 1G12 and 6A12, similarly (albeit not so dramatic) as ACE inhibitors do. Therefore, we can consider that the binding of substance P (as well as ACE inhibitors) to ACE induces dissociation of bilirubin and lysozyme from the complex

with ACE, which, in turn, leads to the increase of ACE shedding and elevation of ACE in the blood.

Then, the ACE-lysozyme hypothesis apparently explains the known fact of a very low ACE level in the urine. Blood ACE in healthy individuals originates from endothelial cells[83], mainly from lung endothelial cells, which express ACE at 100% of lung capillaries in contrast to 10-15 % of capillaries of the systemic circulation[5]. Urine has 50-fold less ACE level relative to blood[42,84,85] and is likely originated from the brush border epithelial cells of kidney proximal tubules. Interestingly, ACE expression in lung capillaries is not elevated relative to proximal tubule epithelial cells of kidney or seminiferous tubules epithelial cells[6,86]. The reasonable explanation for this discrepancy is that kidney proximal tubule epithelial cells is that kind of tissue where lysozyme is filtered through glomerulus. Upon metabolization the concentration of lysozyme on the surface of these cells is the highest in the human body. Therefore, we can suggest that ACE on the surface of kidney proximal epithelial cells is fixed in its most stabile conformation, which prevents excessive ACE shedding and explains low ACE concentration in urine.

Additional **REFERENCES** for **Supplementary Information**

66. Anthony, K.S. *et al.* The N domain of human angiotensin I-converting enzyme: the role of N-glycosylation and the crystal structure in complex with an N domain specific phosphinic inhibitor RXP407. *J. Biol. Chem.* **285**, 35685-35693 (2010).
67. Jaspard, E., Alhenc-Gelas, F. Catalytic properties of the two active sites of angiotensin I-con-verting enzyme on the cell surface. *Biochem. Biophys. Res. Commun*. **211,** 528-534 (1995).
68. Trombetta, E.S., Ebersold, M., Garrett, W., Pypaert, M. & Mellman, I. Activation of lysosomal function during dendritic cell maturation. *Science* **299**, 1400-1403 (2003).
69. Arcasoy, S.M., Gondor, J.D.M., Pitt, B.R. & Pilewski, J.M. Polycations increase the efficiency of adenovirus-mediated gene transfer to epithelial and endothelial cells in vitro. *Gene Ther.* **4**, 32-38 (1997).
70. Cross, M., Mangelsdorf, I., Wedel, A. & Renkawitz, R. Mouse lysozyme M gene: isolation, characterization, and expression studies. *Proc. Natl. Acad. Sci. USA* **85**, 6232-6236 (1988).
71. Ganz, T. *et al.* Increased inflammation in lysozyme M-deficient mice in response to *Micrococcus luteus* and its peptidoglycan. *Blood* **101**, 2388–2392 (2003).
72. Kase, R., Hazato, T., Kiuchi, Y. & Katayama, T. Species differences in the angiotensin converting enzyme activity of mammalian serum. *Jikken Dobutsu* **35**, 107-108 (1986).
73. Ibarra-Rubio, M.E., Pena, J.C. & Pedraza-Chaverri, J. Kinetic and inhibitory characteristics of serum angiotensin-converting enzyme from nine mammalian species. *Comp. Biochem. Physiol. B* **92**, 399-403 (1989).

74. Lieberman, J., Sastre, A. An angiotensin-converting enzyme (ACE) inhibitor in human serum. Increased sensitivity of the serum ACE assay for detecting active sarcoidosis. *Chest* **90**, 869-875 (1986).

75. Wei, L., Alhenc-Gelas, F., Corvol, P. & Clauser, E. The two homologous domains of human angiotensin I-converting enzyme are both catalytically active. *J. Biol. Chem*. **266**, 9002-9008 (1991).

76. Petrov, M.N. *et al.* Conformational changes of blood ACE in chronic uremia. *PLoS One* **7**, e49290, doi: 10.1371/journal.pone.0049290 (2012).

77. Alpert, A.J., Shukla, A.K. Precipitation of large, high-abundance proteins from serum with organic solvents. The 8th Annual Meeting of the Association for Biomedical Resource Facilities (ABRF-2003), Denver, CO, USA, Poster P111-W (2013).

78. Bye, J.W.,Cowieson, N.P., Cowieson, A.J., Selle, P.H. & Falconer, R.J. Dual effects of sodium phytate on the structural stability and solubility of proteins.*J. Agric. Food Chem.* **61**, 290-295 (2013).

79. Zhang, H-M., Xu, Y-Q., Zhou, Q-H. & Wang, Y-Q. Investigation of the interaction between chlorophenoles and lysozyme in solution. *J. Photochem. Photobiol*. **104**, 405-413 (2011).

80. Dumoulin, M., Johnson, R.J.K, Bellotti, V. & Dobson, C.M. Human lysozyme. In *Protein Misfolding, Aggregation and Conformational Diseases. II. Molecular basis of Conformational Diseases*. (Eds. Uversky, V.N., and Fink, A.L.) 285-308, (Kluwer Academic / Plenum Publishers, Dordrecht, Netherlands, 2007).

81. Linnebank, M. *et al.* Hereditary elevation of angiotensin-converting enzyme suggesting neurosarcoidosis. *Neurology* **61**, 1819-1820 (2003).

82. Skidgel, R.A., Engelbrecht, S., Johnson, A.R. & Erdös, E.G. Hydrolysis of substance p and neurotensin by converting enzyme and neutral endopeptidase. *Peptides* **5**, 769-776 (1984).

83. Ching, S.F., Hayes, L.W. & Slakey, L.L. Angiotensin-converting enzyme in cultured endothelial cells. Synthesis, degradation and transfer to culture medium. *Arteriosclerosis* **3**, 581-588 (1983).

84. Kokubu, K., Kato, I., Nishimura, K., Hiwada, K. & Ueda, E. Angiotensin I-converting enzyme in human urine. *Clin. Chem. Acta* **89**, 375-379 (1978).

85. Baudin, B., Beneteau-Burnat, B., Baumann, F.C. & Giboudeau, J. A reliable radiometric assay for the determination of angiotensin I-converting enzyme activity in urine. *J. Clin. Chem. Clin. Biochem.* **28**, 857-861 (1990).

86. Metzger, R. *et al.* Distribution of angiotensin I-converting enzyme (CD 143) in the normal human kidney and in non-neoplastic kidney diseases. *Kidney Int.* **56,** 1442-1454 (1999).

Table S1. Peptides sequences identified in fractions 33-34.

№	Peptide	RT	Observed m/z	Calculated mass
1	KAVQELGLNAELVK	42,46	757,4	1512,8
2	NSLLNAAAGGATEKG	38,56	711,3	1420,6
3	FFNPVGNEGAENLRLA	49,01	882,4	1762,8
4	PSGGAFGNTLVEVK	47,35	688,3	1374,7
5	AKVPAASWPFVVCTY	53,99	819,9	1637,8
6	YFQFCEGDPKALL	43,04	782,3	1562,7
7	PCTAFNVVYALK	47,9	663,8	1325,6
8	FLNSLTLKAMA	49,08	613,3	1224,6
9	VKPAASWPFVVGYD	53,11	784,4	1566,7
10	LSGLLYEEKP	39,44	590,8	1179,6
11	KVANSNLNAAAGQTSKM	42,3	860,9	1719,8
12	FLVDTHPLYKA	46,21	652,3	1302,7
13	NLPAASWPFVVGYF	53,09	784,4	1566,7
14	NPPAASWPFVVNRP	54,1	784,4	1566,8
15	LLKPFNR	29,26	452,3	902,5
16	VGLSGALKAAY	44,37	533,3	1064,6
17	VGLSGALKAAY	44,51	533,3	1064,6
18	RNLALLTTF	46,23	524,8	1047,6
19	KAALALDVTF	46,25	524,8	1047,6
20	LNPGKAVRYA	39,57	552,8	1103,6
21	NPLGKAVRFA	39,58	552,8	1103,6
22	FLVNTHPLFAK	46,22	652,3	1302,7
23	LRNVSTGN	17,79	431,2	860,4
24	RLNVSTGN	17,85	431,2	860,4
25	VAEQLGLDAELVK	46,73	693,3	1384,7
26	AVEELGLDAQLVK	46,72	693,3	1384,7
27	VFLENVLR	47,15	495,3	988,6
28	VFLENVLR	47,17	495,3	988,6
29	FTRKFK	50,94	421,8	841,5
30	KLLKPLLL	55,76	469,3	936,6
31	QYFTQVD	20,23	450,7	899,4
32	LLALKTF	36,08	411,3	820,5
33	FAAFSMFR	36,53	496,7	991,4
34	WPFPTGPVN	47,09	507,7	1013,5

The 3 kDa filtrate of human citrated plasma was subjected to gel-permeation chromatography on a GE Peptide Superdex column and fractions from chromatography cycles were tested for the effects on mAbs 1G12 and 9B9 binding to serum ACE (as in fig. S19). Fractions 33-34 which increased precipitation of ACE activity by these mAbs were analyzed by LC-MS mass spectrometry. RT-Retention Time; m/z values correspond to double protonated peptides.

Table S2.Possible candidates for LMW ACE effector from human blood.

№		names	hmdb_id		average_ mass	Conc. ug/ml	pKa	Physiol. Charge.
1		Bilirubin	HMDB00054		584,6	14	4,03	-2
2		Heparan sulfate	HMDB00693		637,5	0,5	-	-
3		Myo-inositol hexaphosphate	HMDB03502		660,0	0,34	1.1	-
4		Bilirubin glucuronide	HMDB10332		760,7	64	3,21	-2
5		Bilirubin diglucuronide	HMDB03325		936,9	39	2,97	-2

From over 7000 compounds identified (hmdb_id) in human plasma metabolome[44] (www.hmdb.ca), five compounds met the following criteria: 1) a MW $\leq$ 1 kDa (fig. S13);
2) weak anion (fig. S14); 3) concentration in plasma no less than ACE concentration ~ 450 ng/ml[49], which corresponds to 2.5 nM.
pKa value for myo-inositol hexaphosphate (IP_6) is shown for the first most acidic group (from the range 1.1-2.7). Heparan sulfate resembles heparin by physical properties and is a strong anion.
Both compounds, myo-inositol hexaphosphate and heparan sulfate, are expected to have the physiological charge about -6.

LEGENDS to FIGURES

Figure S1. Identification of the novel ACE mutation.

A. Immunological characterization of the blood ACE from patient # 38.

Conformational fingerprinting of serum ACEs with a set of mAbs to ACE. Sixteen monoclonal antibodies were used to precipitate ACE from sera. Immunoprecipitated ACE activity is presented as a normalized value ("binding ratio"), to highlight differences in immunoprecipitation pattern ("conformational fingerprint") among ACE variants from patient # 38 and carriers of the Y465D mutation (which was expressed as 100%). As a reference (control) we used not a serum from individual with normal ACE, but serum from carriers of Y465D mutation[13], having elevated (6-fold) levels of ACE in the blood. In that case the dilution of the serum for performing comparison of ACE conformational fingerprint from index patient (#38) is the same as dilution of serum from control sample and would not interfere with an assay (see Fig. 2). Data presented as a mean of 3 independent determinations. Colored bars indicates that the differences in ratios are statistically significantly different ($p<0.05$): increased more than 20% - bars highlighted with orange or decreased more than 20% - bars highlighted with yellow.

B. Organization of the identified mutations in ACE gene.

Diagram shows intron-exon structure of human ACE gene and localization of the ACE mutations. The following ACE mutations causing elevated blood ACE were described: Y465D in 8th exon[13], W1197X[11] and P1199L[10] in 25th exon and IVS25+1G>A[12]. Heterozygous mutation Arg532Trp (R532W) in 11th exon was revealed in index patient (#38) by the sequencing PCR product containing this exon of ACE gene. The 573 bp DNA fragment was amplified by PCR with primers Ex11_CP29-CP30_R532W_Fw (TCACACCCTCAATCCACTTCTC, intron 10) and Ex11_CP29-CP30_R532W_Rv (ATTTGTGTCGCCCCATGCCAG, intron 11) using

genomic DNA from subject #38 and corresponding control.R532W substitution could be identified also (instead of sequencing) by the restriction analysis of this 573 bp PCR product with restriction endonucleases *AgeI*. The 573 bp DNA fragment was amplified by PCR with primers indicated above using genomic DNA from subject with R532W mutation (and corresponding control). The restriction endonucleases, mentioned in Table 1, cut the 573 bp PCR product from individual with native, wild-type ACE, into 3 fragments (Table).

Table. Digestion of the 573 bp fragment of WT ACE genomic DNA.

**Enzyme**	**Digestion site**	**1st fragment**	**2nd fragment**	**3nd fragmen**
AgeI	ACCGGT	347 nt	226 nt	
BsaWI	WCCGGW	347 nt	27 nt	199 nt

The mutation R532W eliminates the restriction site for *AgeI* giving an additional 573 undigested PCR product in the case of restrictase AgeI and also eliminate 1st restriction site for *BsaW1* mentioned in this Table giving 2 fragments (374 and 199 bp) instead of three fragments in the case of restrictase BsaW1.

Fig. S2. Immunological characterization of the human recombinant mutant (R532W) ACE. Conformational fingerprinting of mutant ACE. Transmembrane anchor containing (**A**) (lysates from corresponding CHO cells) and soluble (**B**) WT and mutant ACE were normalized to achieve 5 mU/ml ACE activity with Hip-His-Leu as a substrate and incubated in microtiter plate wells covered with 16 mAbs to human ACE as in fig. S1A. Data (mean ± SD of 2-3 independent experiments in duplicate) are expressed as ratio of ACE activity precipitated by mAbs from mutant ACE to that of WT ACE. Bars highlighted with yellow and blue – the values of precipitated ACE activity from mutant ACE were more than 20% and 50% lower (respectively),

than corresponding values for WT. Bars highlighted with orange and red - the values of precipitated ACE activity from mutant ACE were more than 20% and 100% higher, respectively, than these values for WT. Colored bars indicate that the differences in ratios are statistically significantly different ($p<0.05$):

Figure S3. Effect of sera on ACE expression and ACE shedding.

ACE-expressing cells: CHO-ACE (clone 2C2[54], HEK-ACE cells[20] and Human Umbilical Vein Endothelial Cells (HUVEC, not older than 5th passage[29]) expressing recombinant human WT ACE (CHO and HEK) and natural human ACE (HUVEC) were cultivated in 6-well plate dishes and incubated with serum-free medium (SFM) - DMEM for CHO and HEK cells and EBM-2 [Lonza, Walkersville, MD] for HUVEC, or with these media containing 10% of FBS or Human Serum (HS) or with heat-inactivated (HI, 65^{0}C, 30 min) counterparts, for 24 hours. Then, culture medium (2 ml) was collected and lysates from washed cell monolayers were prepared (0.5 ml) using 0.25% Triton X-100 as detergent. Cell-bound and soluble ACE activity was determined fluorimetrically with Z-Phe-His-Leu as a substrate. (Mean ± SD of four experiments performed in du- or triplicates).

A. Transmembrane anchor containing ACE activity (lysate). Data are expressed as a percentage from the mean of WT ACE in each cell type, which absolute values of ACE activity were also shown. **B**. Soluble ACE activity. **C.** Rate of ACE secretion. The rate of ACE cleavage was determined as the ratio between ACE in the culture medium and the total amount of cell associated and soluble ACE activity. Data are expressed as a percentage from that of WT ACE in each cell type (B, C), and the absolute values for the rate of shedding are also shown. Bars coloring – as in fig.S2.

Figure S4. The rate of mutant ACE shedding. Effect of serum and ACE inhibitor enalaprilat. CHO cells stably transfected with WT ACE and with R532W mutant as in[13], were cultivated in 6-well plate dishes and incubated with serum-free medium (SFM) - DMEM or DMEM containing 10% of heat-inactivated (65^{0}C, 30 min) FBS or human serum (HS) for 24 hours. Then, culture medium (2 ml) was collected and lysates from washed cell monolayers were prepared (0.5 ml) using 0.25% Triton X-100 as detergent. Cell-bound and secreted ACE activity was determined fluorimetrically with Z-Phe-His-Leu as a substrate. (Mean ± SD of four experiments performed in du- or triplicates). The rate of ACE shedding was determined as the ratio between ACE in the culture medium and the total amount of cell associated and secreted ACE activity. Data are expressed as a percentage from the mean of WT ACE for each type of culture medium. Bars coloring – as in fig.S2.

A. Effect of R532W mutation; **B**. Effect of enalaprilat (100 nM).

Figure S5. Lysozyme binds to ACE.

A. Plate Precipitation assay. Wells of the microtiter plate were coated with BSA, human β2-microglobulin, human recombinant lysozyme (expressed in rice), or mAb i2H5 to ACE at 10 ug/ml. After washing of unbound proteins by PBS-Tween 20 (0.05%) wells were incubated with different dilutions of normal pooled human serum. After washing (once) wells were incubated with ACE substrate (Hip-His-Leu, 5 mM, overnight at 37^{0}C) and precipitated ACE activity was estimated directly in the wells[50]. Data are expressed as a fluorescence arbitrary units, reflecting precipitated ACE activity. Data are mean ± SD of triplicates. * - $p<0.05$ in comparison with mean value for control samples. Insert: results for 1/2.5 plasma dilution showed at higher scale.

B. Surface Plasmon Resonance (BIACORE). Purified human lung ACE was immobilized on sensor via mAb 9B9. Lysozyme or beta2-microglobulin was injected (arrow) at concentration of 10 nM.

Figure S6. Effect of lysozyme on ACE conformation (mAbs binding).

The effect of human lysozyme (1 mg/ml) on mAbs binding to soluble truncated recombinant N- and C-domains of human ACE (**A**) or to recombinant somatic ACE bearing membrane anchor (lysate, **B**), all expressed in CHO cells, was determined using plate precipitation assay as in fig. S2. Data are mean ± SD of 3-4 independent experiments (each in duplicates).

Data are presented as a ratio of ACE activity precipitated by a given mAb in the presence and absence of human lysozyme. Bars highlighted with red indicates that the ratio shown is significantly different (increased) more than 20% ($p<0.05$) in comparison with corresponding value for samples without lysozyme.

Figure S7. Detection of human lysozyme of the surface of CHO-ACE cells by flow cytometry.

CHO cells expressing testicular (CHO-tACE) and somatic (CHO-ACE) isoforms of ACE were pre-incubated overnight with human lysozyme or BSA (as negative control) at 250 ug/ml (as in Fig. 4). Then cells were detached from Petri dish by cold EDTA solution, washed in PBS, incubated subsequently with anti-human lysozyme mAb (or anti-ACE mAb 4E3 as a positive control) and then with anti-mouse IgG conjugated with FITC in the presence of propidium iodide. Fluorescence bound to the surface of alive cells was quantified by flow cytometry using LSRFortessa (Becton-Dickinson, Franklin Lakes, NJ). Data from several representative

histograms (**A**) are shown as a percentage of lysozyme- or ACE-positive cells, i.e. % of cells having fluorescence higher than fluorescence of cells incubated with non-immune mouse IgG after the subtraction of background fluorescence from each histogram. (**B)** Detection of lysozyme-and ACE-positive cells among CHO-ACE cells. (**C-D**) Detection of lysozyme-positive cells. (**D**). CHO-ACE cells pre-incubated with lysozyme were analyzed by flow cytometry with anti-lysozyme Abs after pretreatment with BSA or protamine sulfate. Bars coloring is as in fig. S2.

Figure S8. ACE activity in lysozyme deficient and overexpressing mice.

Mice were sacrificed, blood and tissues weighed, and homogenates (1:6 w/v ratio) prepared as described in Methods. ACE activity was measured fluorimetrically with Hip-Hs-Leu as a substrate.

A. ACE activity in blood (mU/ml) and lung homogenate (mU/ml) from wild-type (WT) mice (FVB/N strain), lysozyme M-deficient mice (Lys $M^{-/-}$), lysozyme M- and P-deficient mice (Lys MP-/-) and mice overexpressing rat lysozyme in lung epithelial cells (Lys $^{+++}$).

B. ACE activity in different organs of 4 week old Lys M $^{-/-}$ mice expressed as percentage of age-matched WT mice (FBN/Y). Data are mean ± SD, N =3; * $p< 0.05$. Bars coloring as in fig. S2.

Figure S9. Alignment of amino acid sequences of lysozyme from different species.

Amino acid sequences of the lysozymes (mature protein, without signal peptide) were taken from the GenBank database with corresponding accession numbers. Amino acid residues identical for all residues or in the most species (consensus) are highlighted by grey color. Lysozyme amino acid residues that are similar in the four species that exhibit the highest blood ACE levels, and

different from species with the lowest blood ACE levels are highlighted in red and yellow. ACE activity, measured in the serum of these species with Z-Phe-His-Leu as a substrate, is shown on the left column. Four species having highest level of blood ACE activity are highlighted in red.

Fig. S10. Model of somatic ACE with 14 kD ACE-binding model protein.

Human lysozyme (14 kD model protein) was docked to the cleft between N and C domains of the model of full-size, two domain porcine ACE based on electron microscopy (EM) data[59]. Several plausible orientations of this protein in the complex with ACE are shown.

Figure S11. Effect of serum dilution on ACE activity

ACE activity in serum samples was quantified using a spectrofluorometric assay with two substrates, Hip-His-Leu (HHL, 5 mM) and Z-Phe-His-Leu (ZPHL, 2 mM). Serum pooled from 3 healthy donors was diluted by PBS (pH 7.4) and 10 µM $ZnCl_2$. Differently diluted serum (100 µl) or different solutions of soluble recombinant human ACE were then added to 100 µl of substrate solution[42]. The reaction was carried at 37^0C, concentration of the product of enzymatic hydrolysis, His-Leu, was quantified fluorimetrically by reaction with *o*-phthaldialdehyde.

A. Dependence of measurable ACE activity on serum dilution. Dashed line – theoretical ACE activity at different dilutions based on the value of ACE activity in 1/10 dilution of human serum as starting point. **B**. Data are expressed as a percentage of ACE activity calculated for undiluted serum with different serum dilutions. **C**. Data are expressed as the ratio of the rates of the hydrolysis of two substrates -ZPHL/HHL ratio[42] by serum ACE at different dilutions compared with that for recombinant ACE. The results are shown as means + SD of several (2-3) experiments. * - $p<0.05$ in comparison with mean value for all samples.

Figure S12. Effect of serum and serum filtration on mAbs binding to blood ACE.

A-C. Precipitation of ACE activity from plasma samples was performed with 16 mAbs to different epitopes of human ACE as in fig.S1A.

A. Effect of heat–inactivated (65^0C, 30 min) human serum (30%) on ACE activity precipitation by this set of mAbs from human serum diluted 1/10. **B.** Effect of filtrate (10%) after serum filtration through Amicon filter (with 5 kDa limit) on ACE activity precipitation by this set of mAbs from human serum diluted 1/10. **C.** Effect of serum filtration through Amicon filter (with 5 kDa limit) on ACE activity precipitation by this set of mAbs from human serum supernatant retained over the filter. Data are expressed as a percentage of ACE activity precipitated in the presence of heat-inactivated human serum (**A**), or in the presence of filtrate (**B**), or the activity in the supernatant (**C**) from that for control pooled human serum. Data are mean $\pm$ SD of 2-3 independent experiments each performed in duplicates. Bars coloring as in fig. S2.

Figure S13. Effect of dialysis or serum filtration on mAbs binding to blood ACE.

A-D. Precipitation of ACE activity from plasma samples which were dialysed or filtered through different pores was performed as in Fig.1A.

Effect of dialysis through 10 kDa (**A**), 3.5 kDa (**C**) and 1 kDa (**D**) pores and filtration through 5 kDa pores (**B**) on ACE activity precipitation by this set of mAbs from human serum diluted 1/5. Data are expressed as a percentage of ACE activity precipitated after dialysis or filtration to that of control sample. Data are mean $\pm$ SD of 2-3 independent experiment each performed in duplicates. Bars coloring as in fig. S2.

Figure S14. Effect of LMW compounds from serum on mAbs binding to blood ACE.

A-C. Precipitation of ACE activity from plasma samples diluted 1/5 after adding the supernatant of human plasma after ACN precipitation of HMW serum proteins was performed as in fig. S12. Effect of the whole supernatant (10%) (**A**), or supernatant after passing through Sep-Pak column S18 (**B**) or through weak anion exchanger spin column D (**C)** was expressed as a percentage of ACE activity precipitated by mAb in the presence of tested supernatants to that of control sample. Data are mean $\pm$ SD of 2-3 independent experiment each performed in duplicates.

Figure S15. Effect of sera, bovine serum albumin and lysozyme on ACE shedding *in vitro*.

Effect of different compounds on ACE shedding *in vitro* was estimated as in fig. S3. The rate of ACE shedding was determined as the ratio between ACE in the culture medium and the total amount of cell associated and secreted ACE activity. Data are expressed as a percentage from the mean of ACE shedding in the presence of 10% human serum (**A**) or shedding in Serum Free Medium (**B**). Human serum (1/2 dilution with PBS) was filtered through 3 kDa and 30 kDa spin filters (Vivaspin, GE HealthCare, Piscataway, NJ), heat-inactivated (at 65^0C, 30 min to inactivate human blood ACE) and added to the cells for 24 hours at 10% concentration in F12 medium. Lysozyme, BSA, as well as BSA, which was filtered as 10 mg/ml solution through the filter with 30 kDa limit, were added to the cells as 1 mg/ml solution in medium F12. Enalaprilat was added in 100 nM concentration.

Figure S16. Effect of 3 kD filtrate of human plasma on mAbs binding to ACE.

Effect of 3 kDa filtrate on the precipitation of ACE activity from human citrated plasma (**A,** 1/5 dilution) or from the solution of pure ACE isolated from seminal fluid (**B**) was performed with

17 mAbs to different epitopes on human ACE as in fig. S1A. Data are expressed as a percentage of ACE precipitation by tested mAb in the presence of 80% of 3 kDa filtrate to that of untreated human ACE. Data are mean $\pm$ SD of triplicates. Colored bars (as in fig. S2) indicate that the differences in ratios are statistically significantly different ($p<0.05$).

Figure S17. Effect of Enalaprilat and EDTA on mAbs binding to ACE.

A-D. Precipitation of ACE activity from human serum (1/5 dilution) samples (**A** and **C**) and culture fluid from CHO-ACE cells containing soluble recombinant human ACE (**B** and **D**) was performed as in fig. S1A. Effect of 100 nM enalaprilat (**A,B**), and 1 mM EDTA (**C, D)** was expressed as a percentage of ACE activity precipitated in the presence of tested compounds to that of control sample. Data are mean $\pm$ SD of 2-3 independent experiment each performed in duplicates. Colored bars (as in fig.S2) indicate that the differences in ratios are statistically significantly different ($p<0.05$):

Figure S18. Effect of chloride depletion on mAbs binding to ACE.

A-C. Precipitation of ACE activity from human serum, 1/5 dilution (**A**), pure lung ACE (**B**), and ACE from seminal fluid, 1/250 dilution (**C**) was performed as in fig.S1A.

Effect of Cl^- depletion was expressed as a percentage of ACE activity precipitated after Cl^- depletion to that of control sample. Data are mean $\pm$ SD of 2-3 independent experiments, each performed in duplicates. Colored bars - as in fig.S2. indicate that the differences in ratios are statistically significantly different ($p<0.05$).

Figure S19. Analysis of LMW ACE effectors from human blood.

A. Gel-filtration chromatography of human plasma 3 kD filtrate. Filtrate of the human citrated plasma (diluted 1/2 in PBS) filtered through Vivaspin 500 (GE HealthCare) filter with 3 kDa limit was applied on the GE Peptide Superdex column (10/300, GE Healthcare) in 50 mM phosphate buffer, pH 7.5, containing 20 mM NaCl and 10 uM $ZnCl_2$, and eluted using the same buffer. Data are presented as Optical Density (mAU) at 215 nm and 280 nm.

B. Effect of gel-filtration chromatography fractions on the binding of mAbs, 1G12 and 9B9, to ACE from 1/5 diluted plasma was estimated as in fig. S1. Data are expressed as % from control (PBS).

A

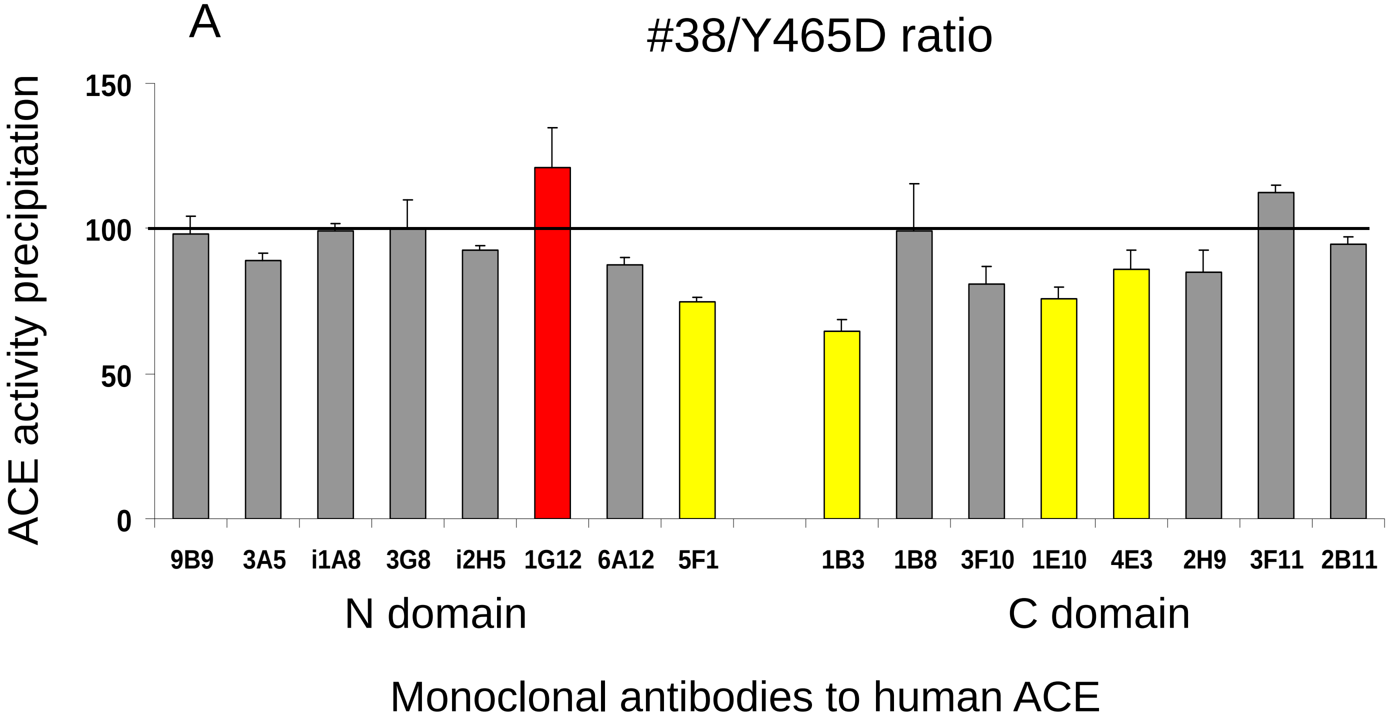

#38/Y465D ratio
ACE activity precipitation
150
100
50
0
9B9
3A5
i1A8
3G8
i2H5
1G12
6A12
5F1
1B3
1B8
3F10
1E10
4E3
2H9
3F11
2B11
N domain
C domain
Monoclonal antibodies to human ACE

B

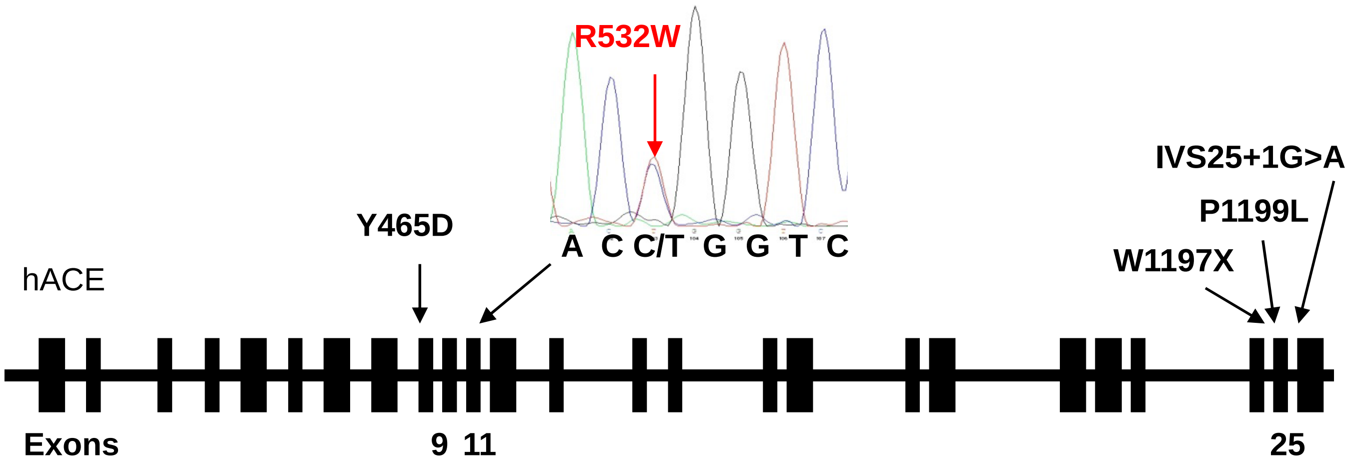

R532W
IVS25+1G>A
P1199L
W1197X
Y465D
A C C/T G G T C
hACE
Exons
9 11
25

Fig. S1

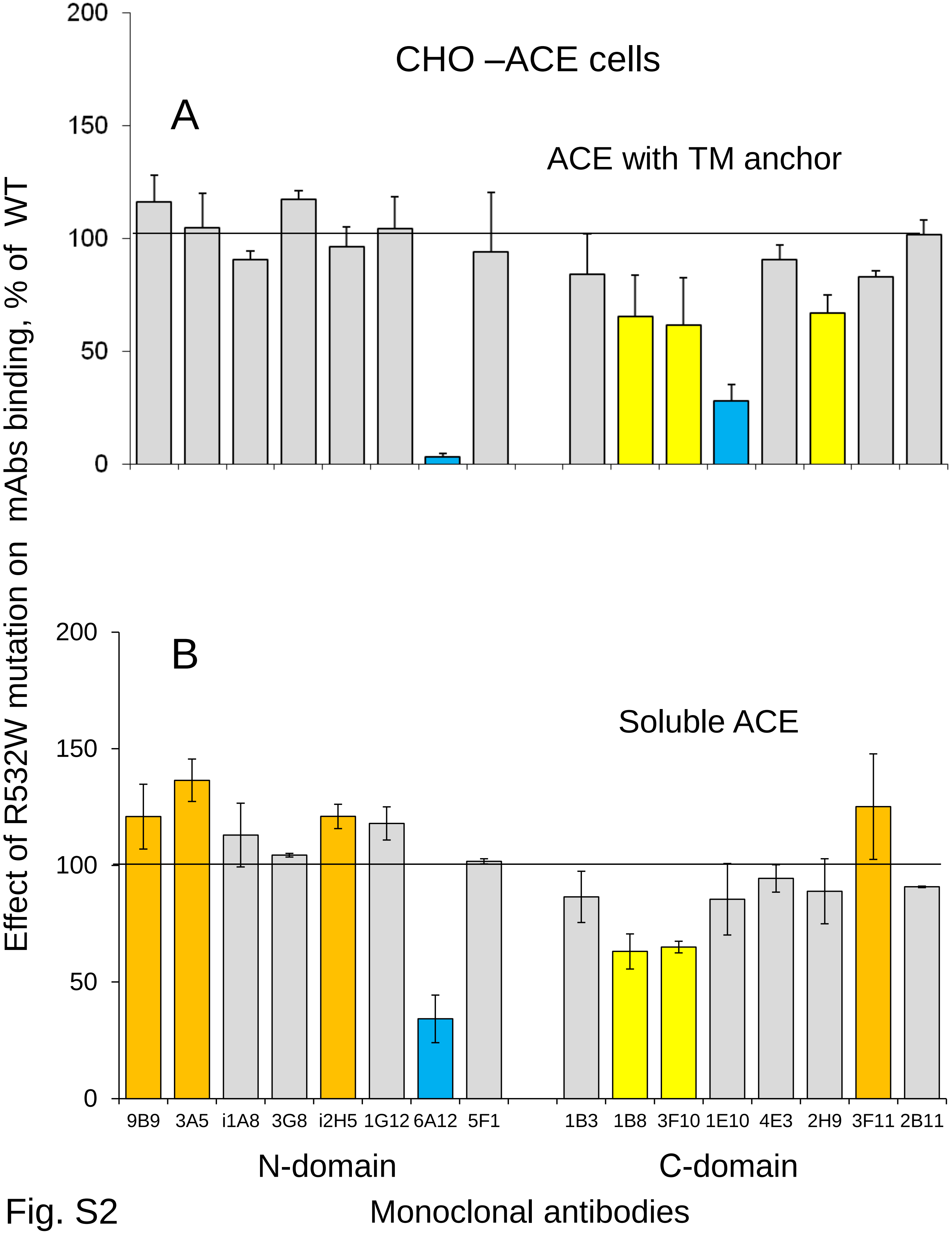
CHO –ACE cells
A
ACE with TM anchor
B
Soluble ACE
Effect of R532W mutation on mAbs binding, % of WT
200
150
100
50
0
9B9
3A5
i1A8
3G8
i2H5
1G12
6A12
5F1
1B3
1B8
3F10
1E10
4E3
2H9
3F11
2B11
N-domain
C-domain
Monoclonal antibodies

Fig. S2

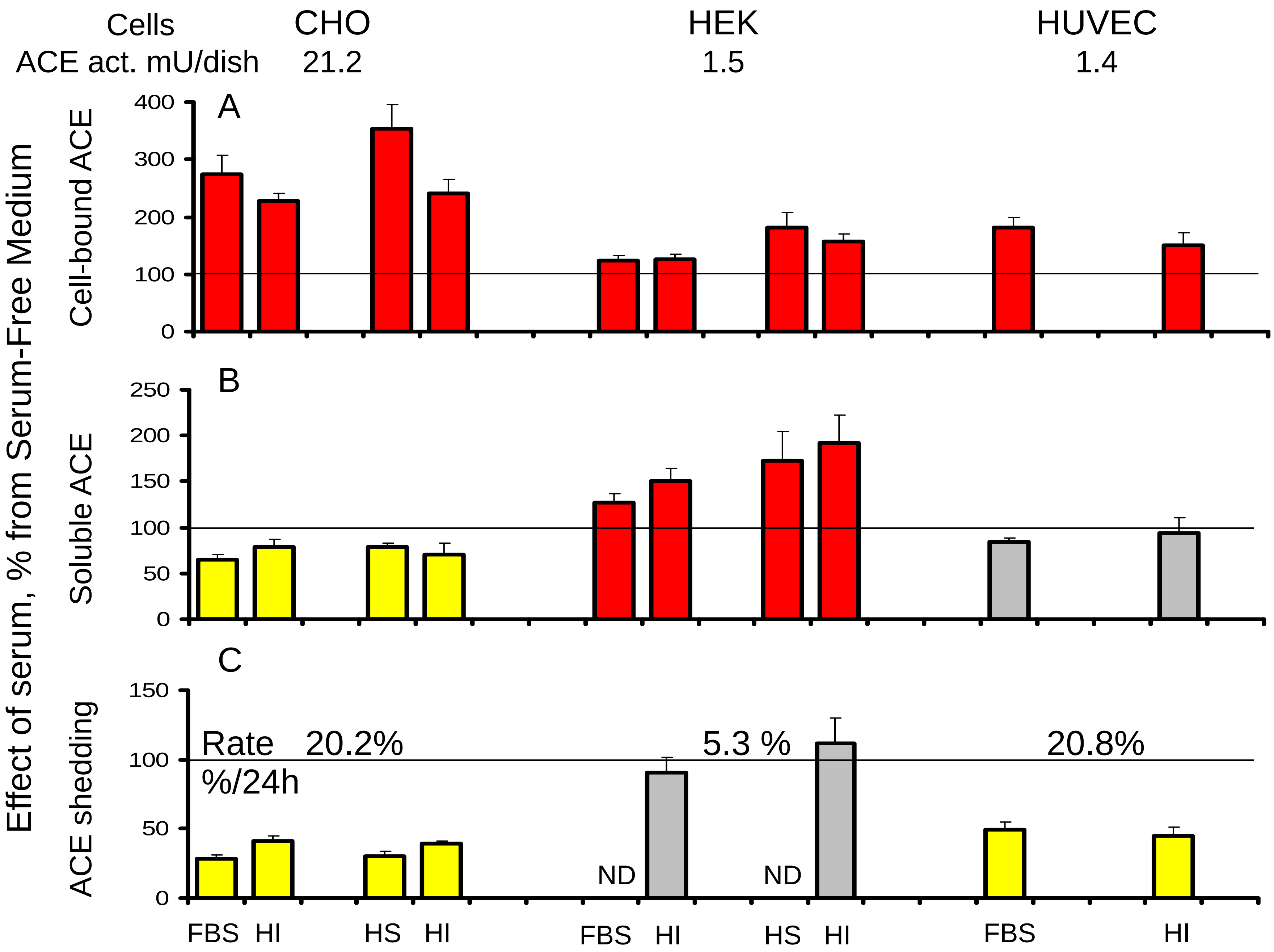
Cells
ACE act. mU/dish
CHO
21.2
HEK
1.5
HUVEC
1.4
Effect of serum, % from Serum-Free Medium
A
Cell-bound ACE
400
300
200
100
0
B
Soluble ACE
250
200
150
100
50
0
C
ACE shedding
150
100
50
0
Rate
%/24h
20.2%
5.3 %
20.8%
ND
ND
FBS
HI
HS
HI
FBS
HI
HS
HI
FBS
HI

Fig. S3

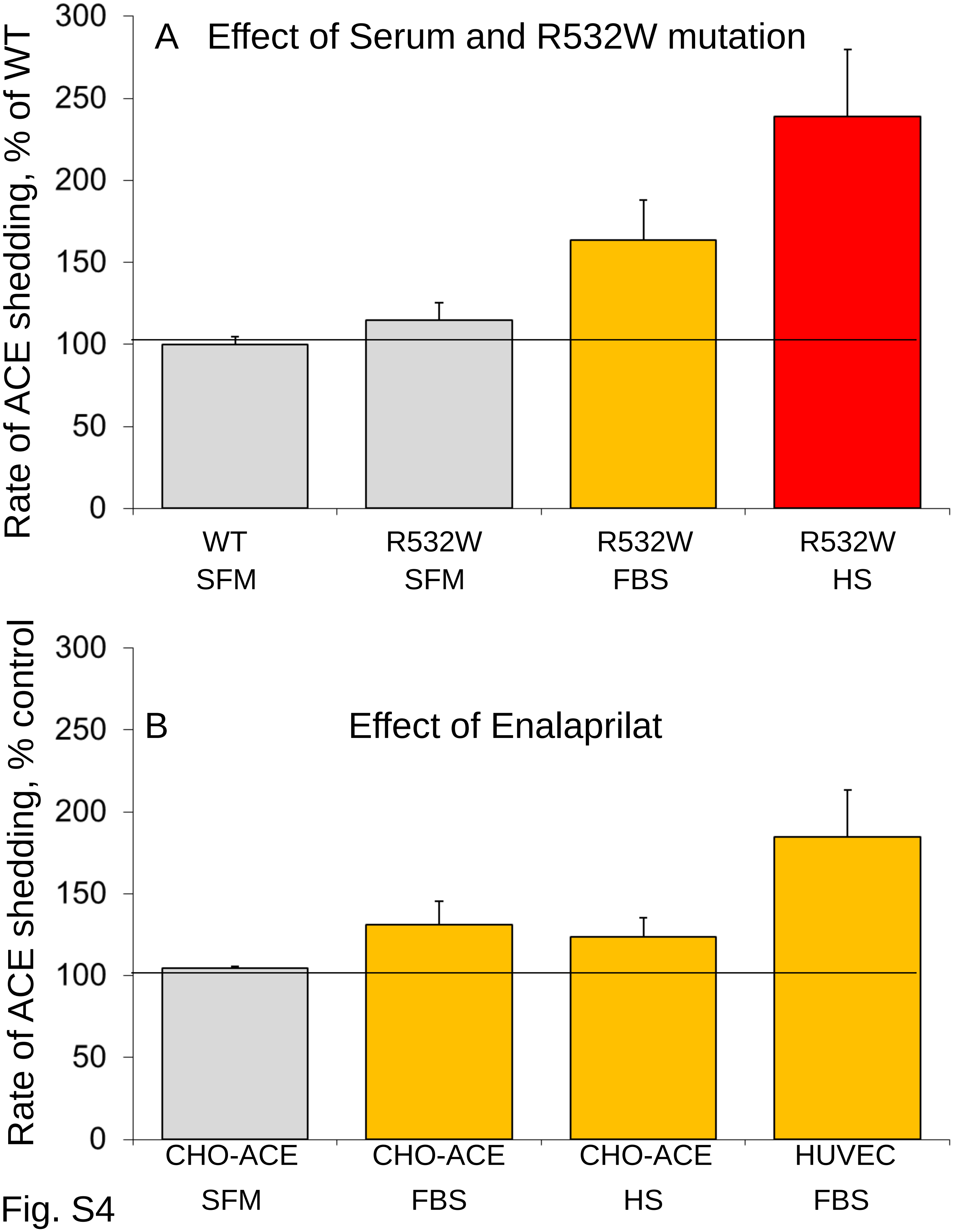
A Effect of Serum and R532W mutation
Rate of ACE shedding, % of WT
300
250
200
150
100
50
0
WT
SFM
R532W
SFM
R532W
FBS
R532W
HS
B
Effect of Enalaprilat
Rate of ACE shedding, % control
300
250
200
150
100
50
0
CHO-ACE
SFM
CHO-ACE
FBS
CHO-ACE
HS
HUVEC
FBS

Fig. S4

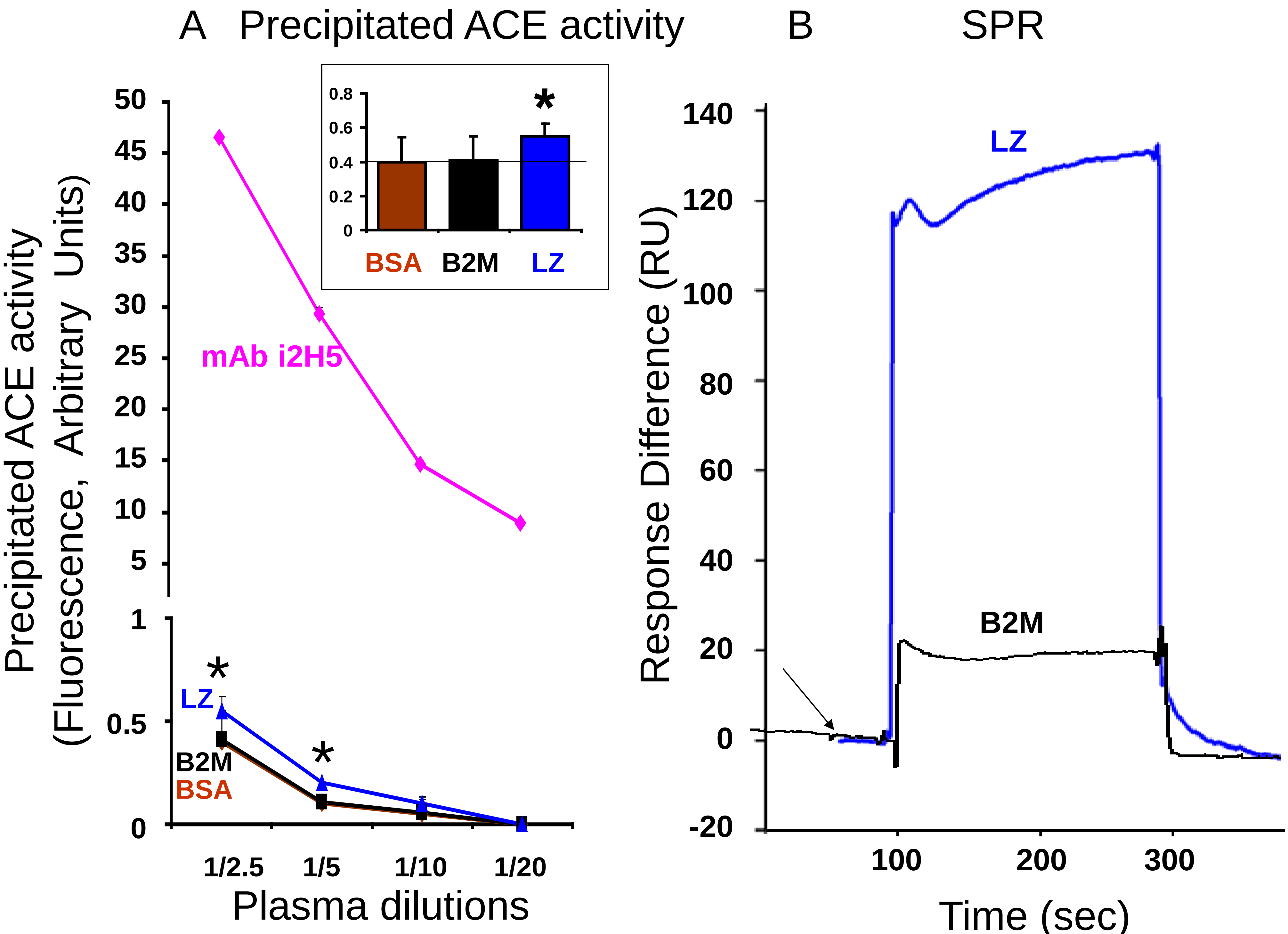
A Precipitated ACE activity
B SPR
Precipitated ACE activity
(Fluorescence, Arbitrary Units)
50
45
40
35
30
25
20
15
10
5
1
0.5
0
mAb i2H5
LZ
B2M
BSA
*
*
1/2.5
1/5
1/10
1/20
Plasma dilutions
0.8
0.6
0.4
0.2
0
BSA
B2M
LZ
Response Difference (RU)
140
120
100
80
60
40
20
0
-20
100
200
300
Time (sec)

Fig. S5

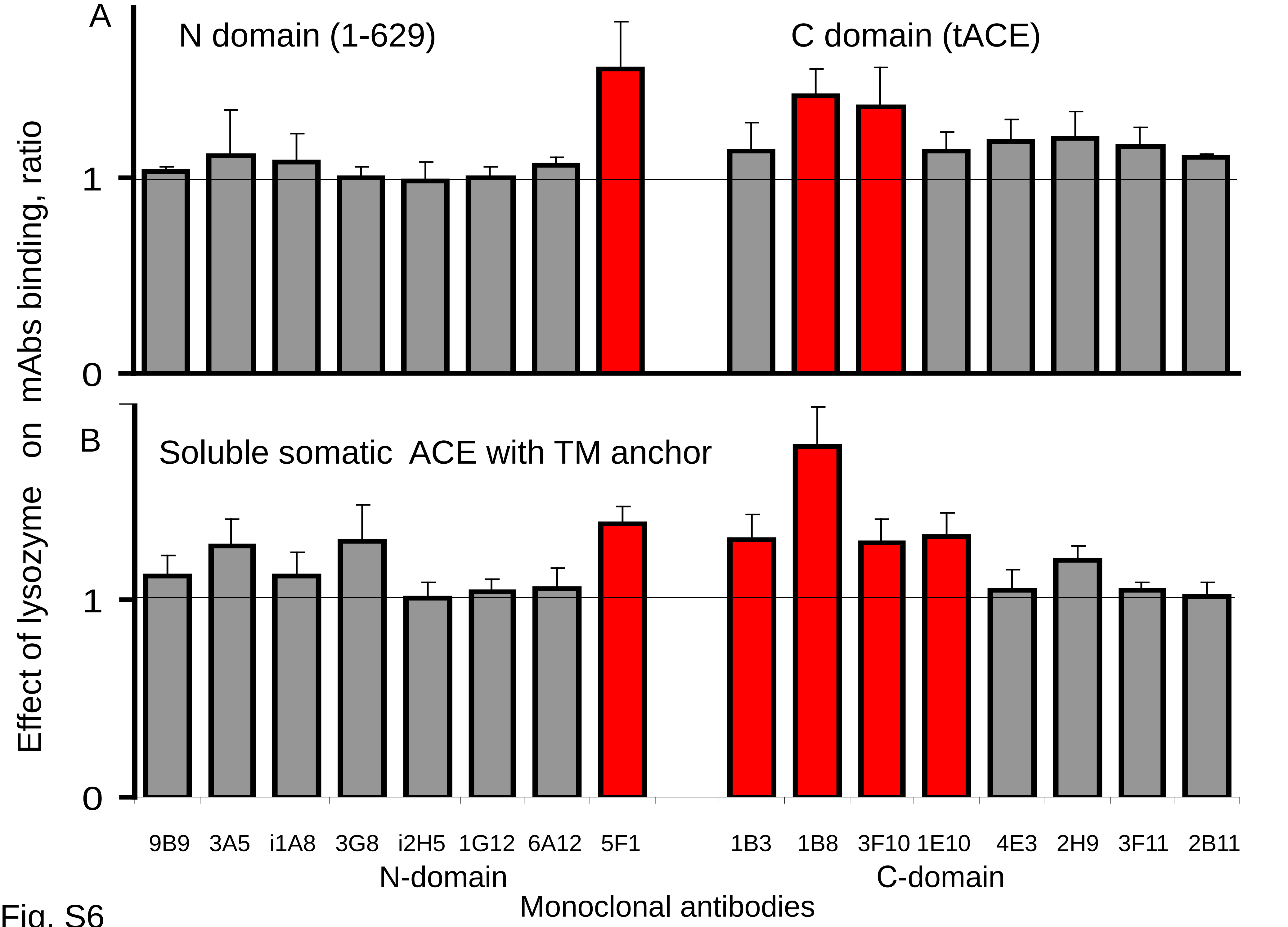

A
N domain (1-629)
C domain (tACE)
B
Soluble somatic ACE with TM anchor
Effect of lysozyme on mAbs binding, ratio
0
1
9B9
3A5
i1A8
3G8
i2H5
1G12
6A12
5F1
1B3
1B8
3F10
1E10
4E3
2H9
3F11
2B11
N-domain
C-domain
Monoclonal antibodies

Fig. S6

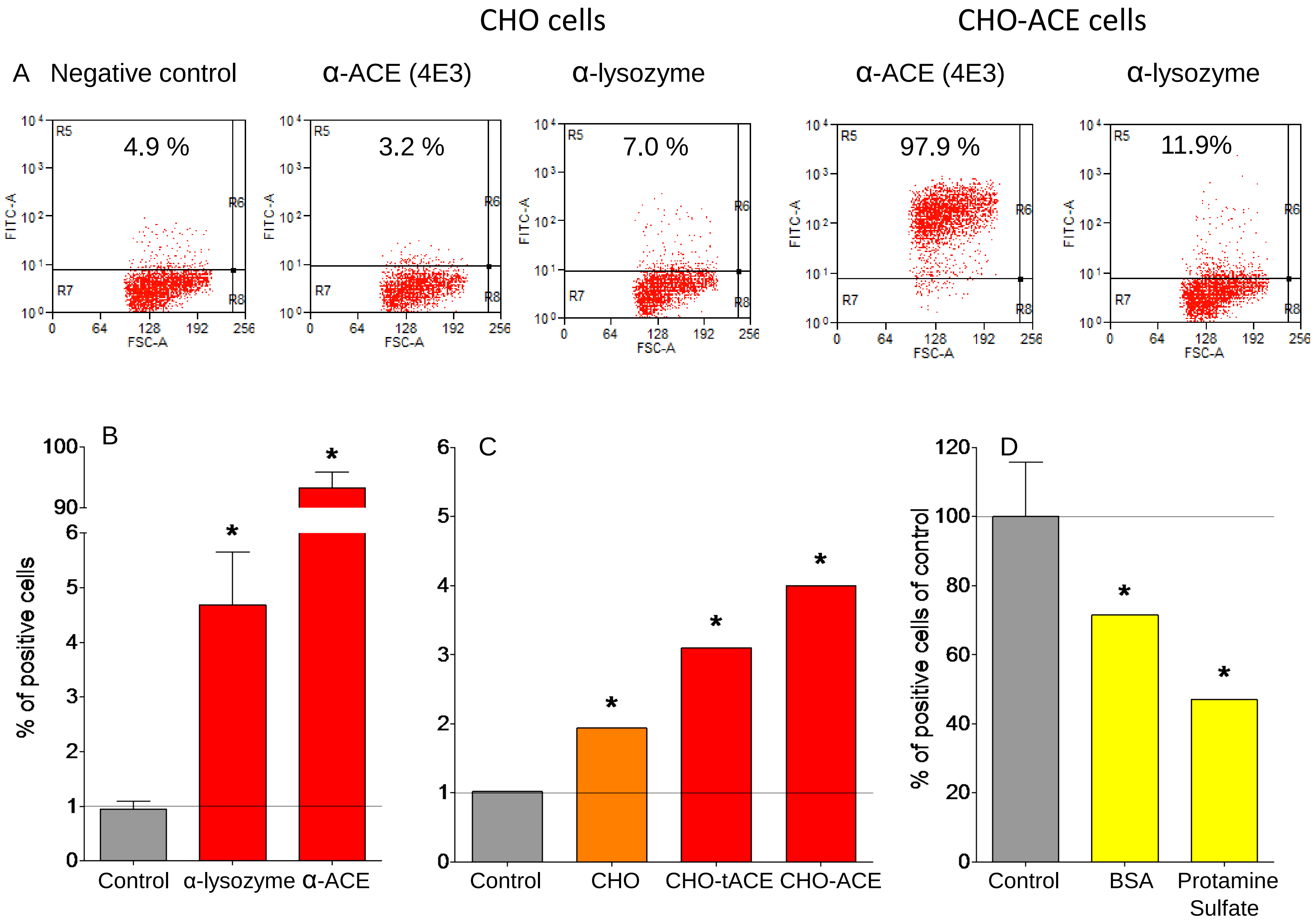
CHO cells
CHO-ACE cells
A Negative control
α-ACE (4E3)
α-lysozyme
α-ACE (4E3)
α-lysozyme
4.9 %
3.2 %
7.0 %
97.9 %
11.9%
R5
R6
R7
R8
FITC-A
FSC-A
B
C
D
% of positive cells
% of positive cells of control
Control
α-lysozyme
α-ACE
Control
CHO
CHO-tACE
CHO-ACE
Control
BSA
Protamine Sulfate

Fig. S7

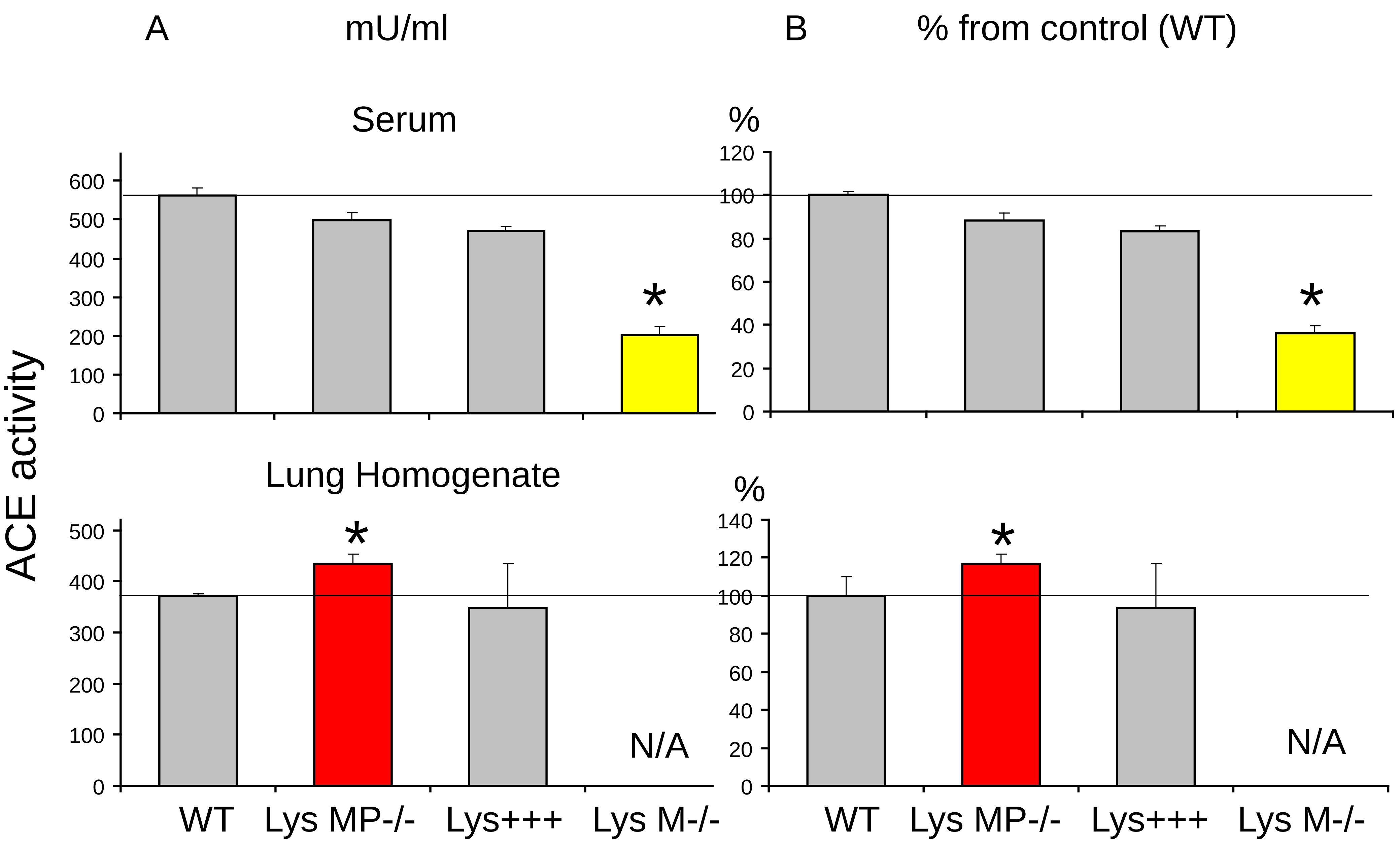
A
mU/ml
B
% from control (WT)
Serum
%
ACE activity
0
100
200
300
400
500
600
0
20
40
60
80
100
120
*
*
Lung Homogenate
%
0
100
200
300
400
500
0
20
40
60
80
100
120
140
*
*
N/A
N/A
WT
Lys MP-/-
Lys+++
Lys M-/-
WT
Lys MP-/-
Lys+++
Lys M-/-

Fig. S8

Serum **ACE** activity mU/ml		**Acces. #**	**Lysozyme sequence** 1	11	21	31	41	51
**681**	Mouse_M	P08905	KVYERCEFAR	TLKRNGMAGY	YGVSLADWVC	LAQHESNYNT	RATNYNRGDQ	STDYGIFQIN
**392**	Rat	P0097	KIYERCQFAR	TLKRNGMSGY	YGVSLADWVC	LAQHESNYNT	QARNYNPGDQ	STDYGIFQIN
**263**	Mouse_P	P17897	KVYNRCELAR	ILKRNGMDGY	RGVKLADWVC	LAQHESNYNT	RATNYNRGDR	STDYGIFQIN
**262**	Swine	P12067	KVYDRCEFAR	ILKKSGMDGY	RGVSLANWVC	LAKWESDFNT	KAINRNVG--	STDYGIFQIN
182	M.Rhe.	P61628	KIFERCELAR	TLKRLGLDGY	RGISLANWVC	LAKWESNYNT	QATNYNPGDQ	STDYGIFQIN
124	Baboon	P61629	KIFERCELAR	TLKRLGLDGY	RGISLANWVC	LAKWESDYNT	QATNYNPGDQ	STDYGIFQIN
114	Bovine	P80189	KVFERCELAR	SLKRFGMDNF	RGISLANWMC	LARWESNYNT	QATNYNAGDQ	STDYGIFQIN
128	Feline	ABH10624	~~~~RCELAR	TLKRLGMDGF	KGVSLATWMC	VAKWESDYNT	RATNYNPGSR	STDYGIFQIN
51	Goat	ACX50258	KVFERCELAR	TLKRFGMDGF	RGISLANWMC	LARWESSYNT	QATNYNSGDR	STDYGIFQIN
40	Sheep	AAB26393	KVFERCELAR	TLKRFGMDGF	RGISLANWMC	LARWESSYNT	QATNYNSGDR	STDYGIFQIN
39	Dog	P81709	KIFERCELAR	TLKNLGLAGY	KGVSLANWVC	LAKWESNYNT	RATNYNPGSK	STDYGIFQIN
39	Rabbit	P16973	KIYERCELAR	TLKKLGLDGY	KGVSLANWMC	LAKWESSYNT	RATNYNPGDK	STDYGIFQIN
29	Human	P61626	KVFERCELAR	TLKRLGMDGY	RGISLANWMC	LAKWESGYNT	RATNYNAGDR	STDYGIFQIN
23	Chimp	P61628	KVFERCELAR	TLKRLGMDGY	RGISLANWMC	LAKWESGYNT	RATNYNAGDR	STDYGIFQIN

		61	71	81	91	101	111	121
**681**	Mouse_M	SRYWCNDGKT	PRAVNACGIN	CSALLQDDIT	AAIQCAKRVV	RDPQGIRAWV	AWRAHCQNRD	LSQYIRNCGV
**392**	Rat	SRYWCNDGKT	PRAKNACGIP	CSALLQDDIT	QAIQCAKRVV	RDPQGIRAWV	AWQRHCKNRD	LSGYIRNCGV
**263**	Mouse_P	SRYWCNDGKT	PRSKNACGIN	CSALLQDDIT	AAIQCAKRVV	RDPQGIRAWV	AWRTQCQNRD	LSQYIRNCGV
**262**	Swine	SRYWCNDGKT	PKAVNACHIS	CKVLLDDDLS	QDIECAKRVV	RDPQGIKAWV	AWRTHCQNKD	VSQYIRGCKL
182	M.Rhe.	SHYWCNNGKT	PGAVNACHIS	CNALLQDNIA	DAVTCAKRVV	SDPQGIRAWV	AWRNHCQNRD	VSQYVQGCGV
124	Baboon	SHYWCNNGKT	PGAVNACHIS	CNALLQDNIA	DAVTCAKRVV	SDPQGIRAWV	AWRNHCQNRD	VSQYVQGCGV
114	Bovine	SHWWCNDGKT	PGAVNACHLP	CGALLQDDIT	QAVACAKRVV	SDPQGIRAWV	AWRSHCQNQD	LTSYIQGCGV
128	Feline	SRYWCNDGKT	PHAENACHVS	CSELLQDNIS	QAVKCAKRVV	SDPQGIKAWV	AWKKHCQNKD	VSQYVRNCGV
51	Goat	SHWWCNDGKT	PGAVNACHIP	CSALLQDDIT	QAVACAKRVV	SDPQGIRAWV	AWRSHCQNQD	LTSYIQGCGV
40	Sheep	SHWWCNDGKT	PGAVNACHIP	CSALLQDDIT	QAVACAKRVV	SDPQGIRAWV	AWRSHCQNQD	LTSYIQGCGV
39	Dog	SRYWCNDGKT	PRAVNACHIS	CSALLQDDIT	QAVACAKRVV	SDPNGIRAWV	AWRAHCENRD	VSQYVRNCGV
39	Rabbit	SRYWCNDGKT	PRAVNACHIP	CSDLLKDDIT	QAVACAKRVV	SDPQGIRAWV	AWRNHCQNQD	LTPYIRGCGV
29	Human	SRYWCNDGKT	PGAVNACHLS	CSALLQDNIA	DAVACAKRVV	RDPQGIRAWV	AWRNRCQNRD	VRQYVQGCGV
23	Chimp	SRYWCNDGKT	PGAVNACHLS	CSALLQDNIA	DAVACAKRVV	RDPQGIRAWV	AWRNRCQNRD	VRQYVQGCGV

Fig. S9

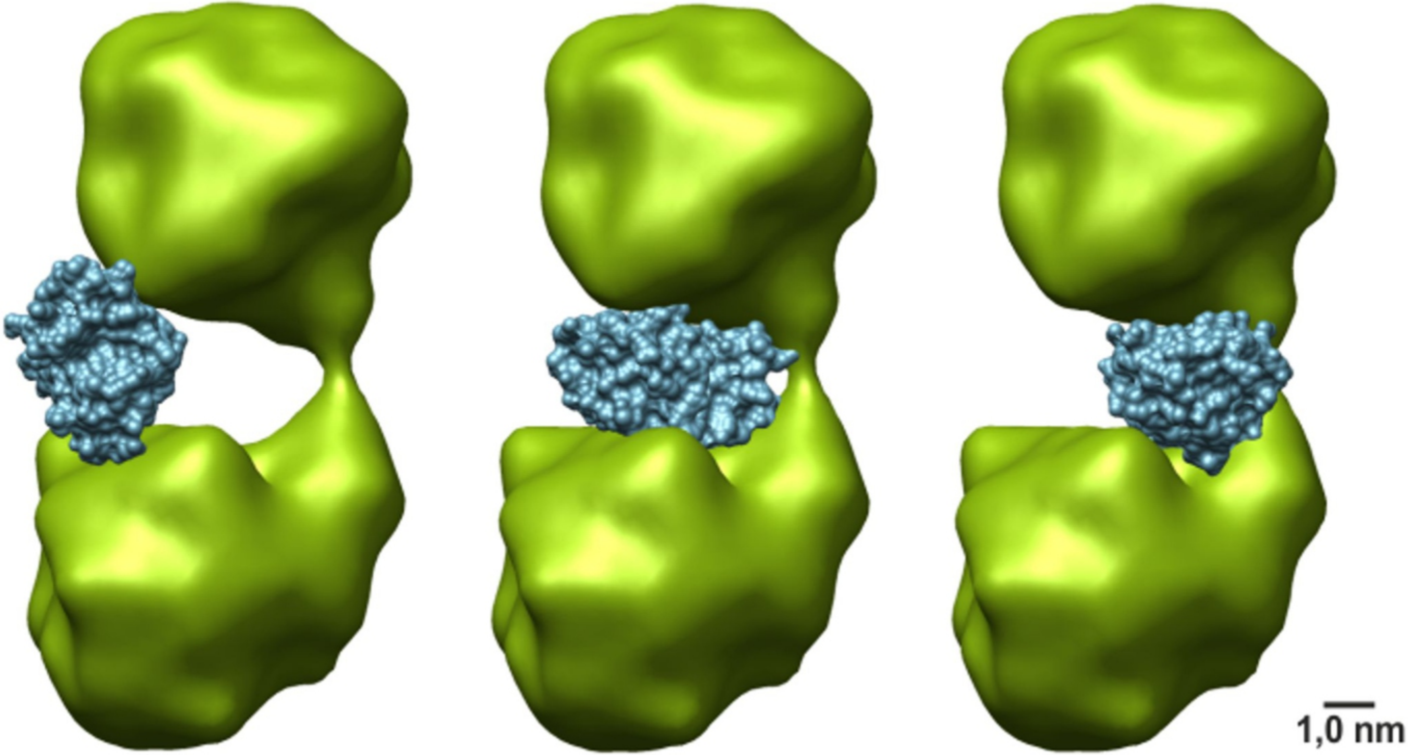

Fig. S10

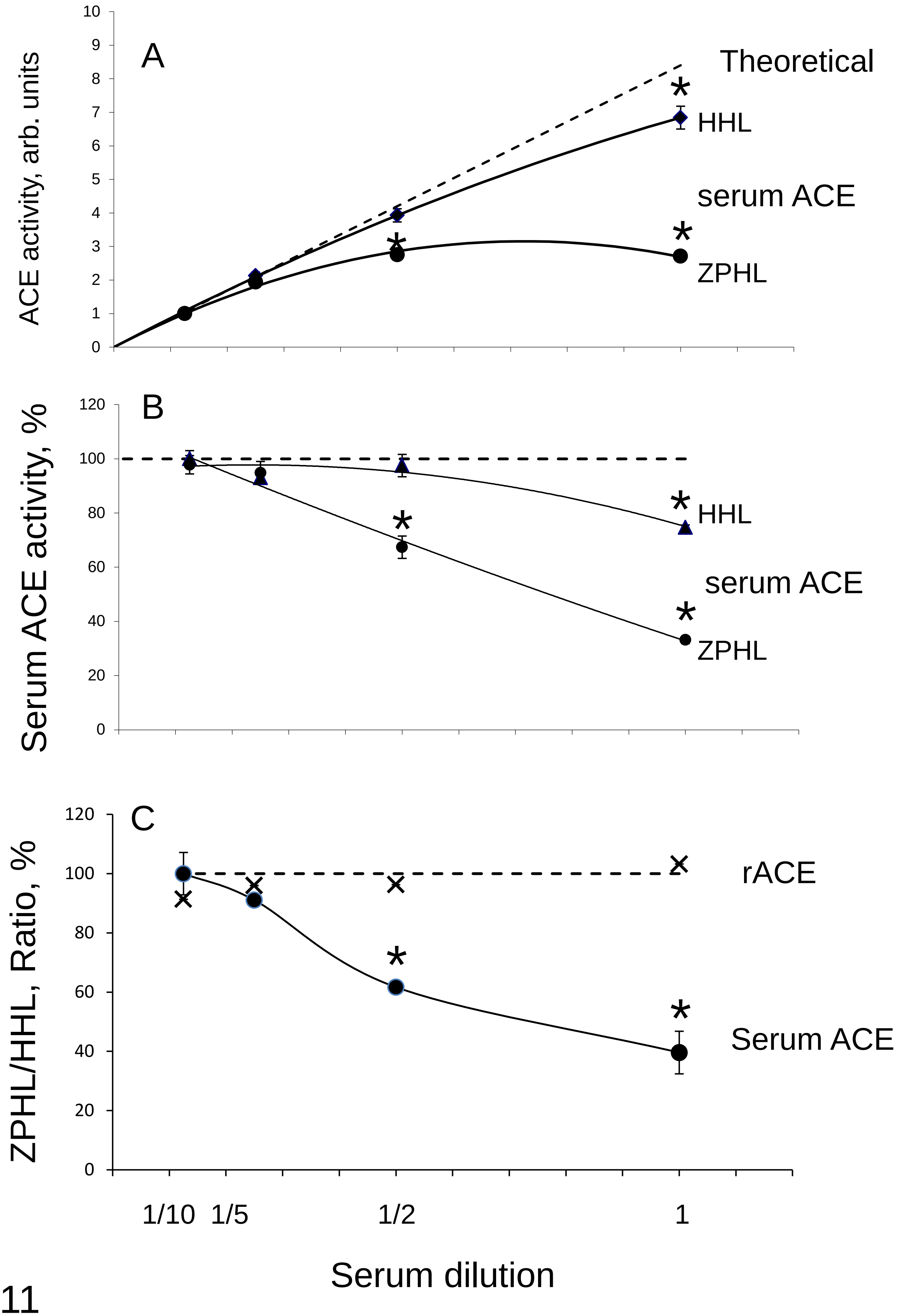
A
Theoretical
HHL
serum ACE
ZPHL
ACE activity, arb. units
B
HHL
serum ACE
ZPHL
Serum ACE activity, %
C
rACE
Serum ACE
ZPHL/HHL, Ratio, %
1/10
1/5
1/2
1
Serum dilution

Fig.S11

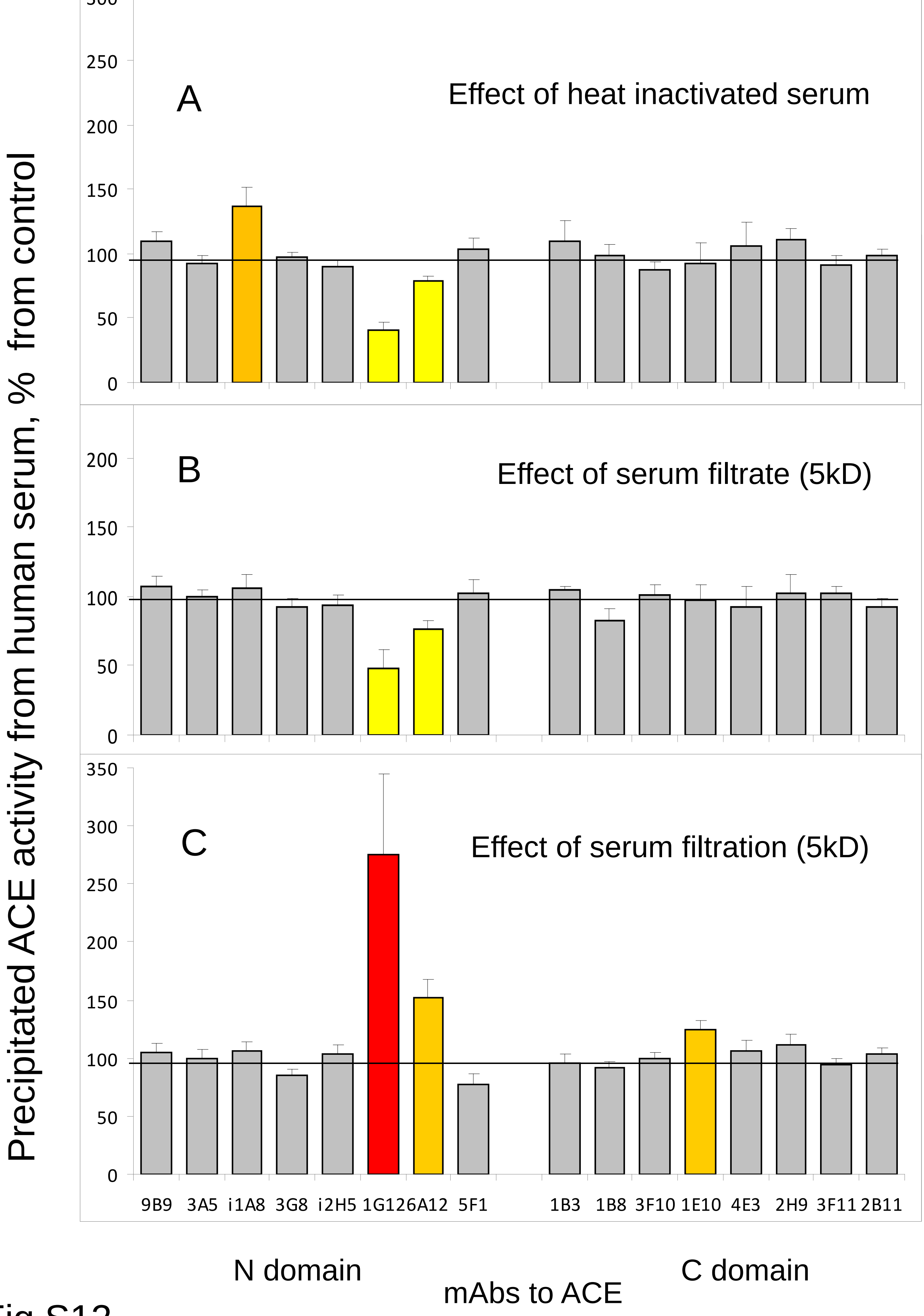

Precipitated ACE activity from human serum, % from control
A
Effect of heat inactivated serum
B
Effect of serum filtrate (5kD)
C
Effect of serum filtration (5kD)
0
50
100
150
200
250
300
350
9B9 3A5 i1A8 3G8 i2H5 1G12 6A12 5F1 1B3 1B8 3F10 1E10 4E3 2H9 3F11 2B11
N domain
C domain
mAbs to ACE

Fig.S12

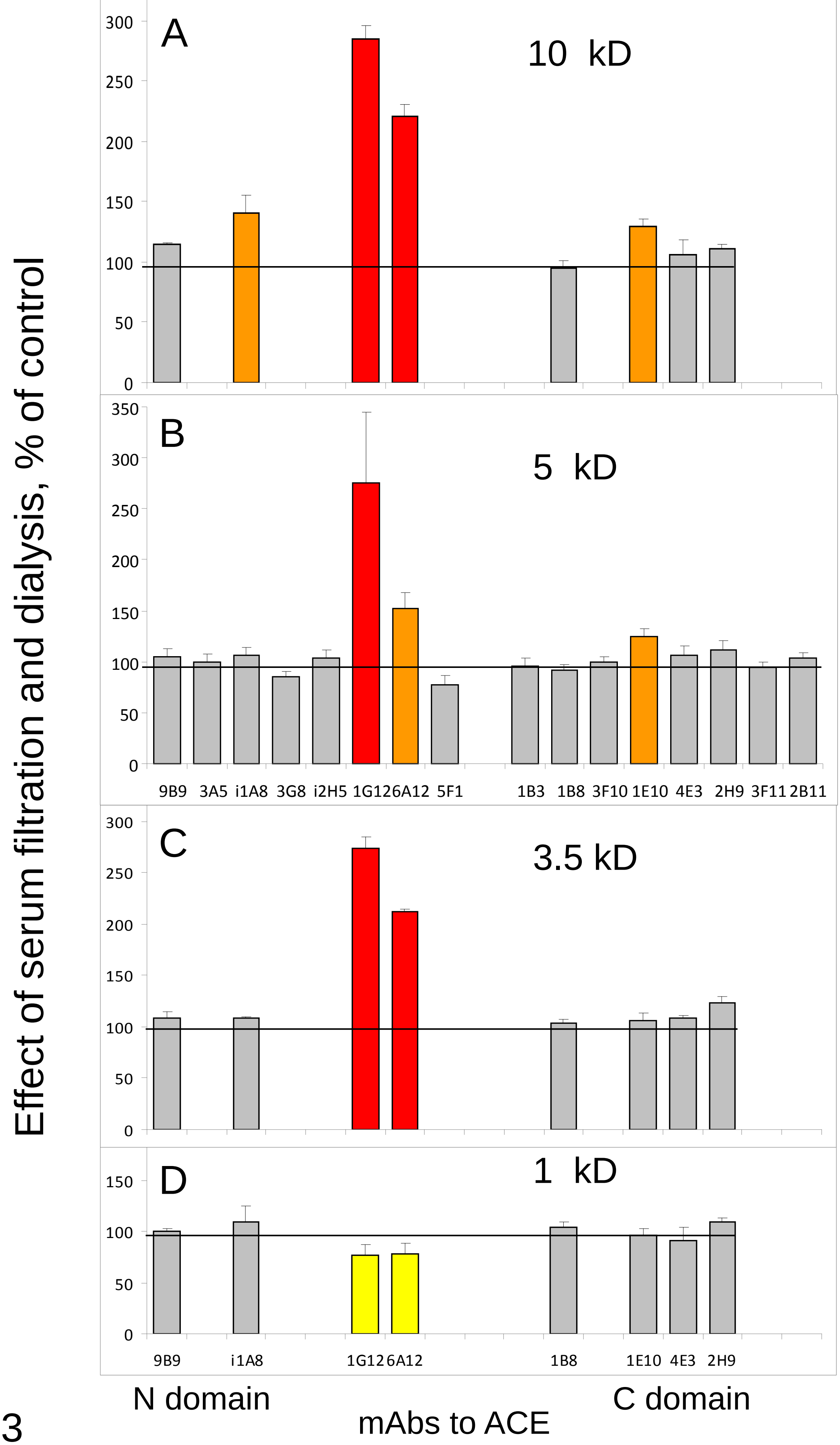
Effect of serum filtration and dialysis, % of control
A
10 kD
B
5 kD
9B9 3A5 i1A8 3G8 i2H5 1G12 6A12 5F1 1B3 1B8 3F10 1E10 4E3 2H9 3F11 2B11
C
3.5 kD
D
1 kD
9B9 i1A8 1G12 6A12 1B8 1E10 4E3 2H9
N domain
C domain
mAbs to ACE

Fig. S13

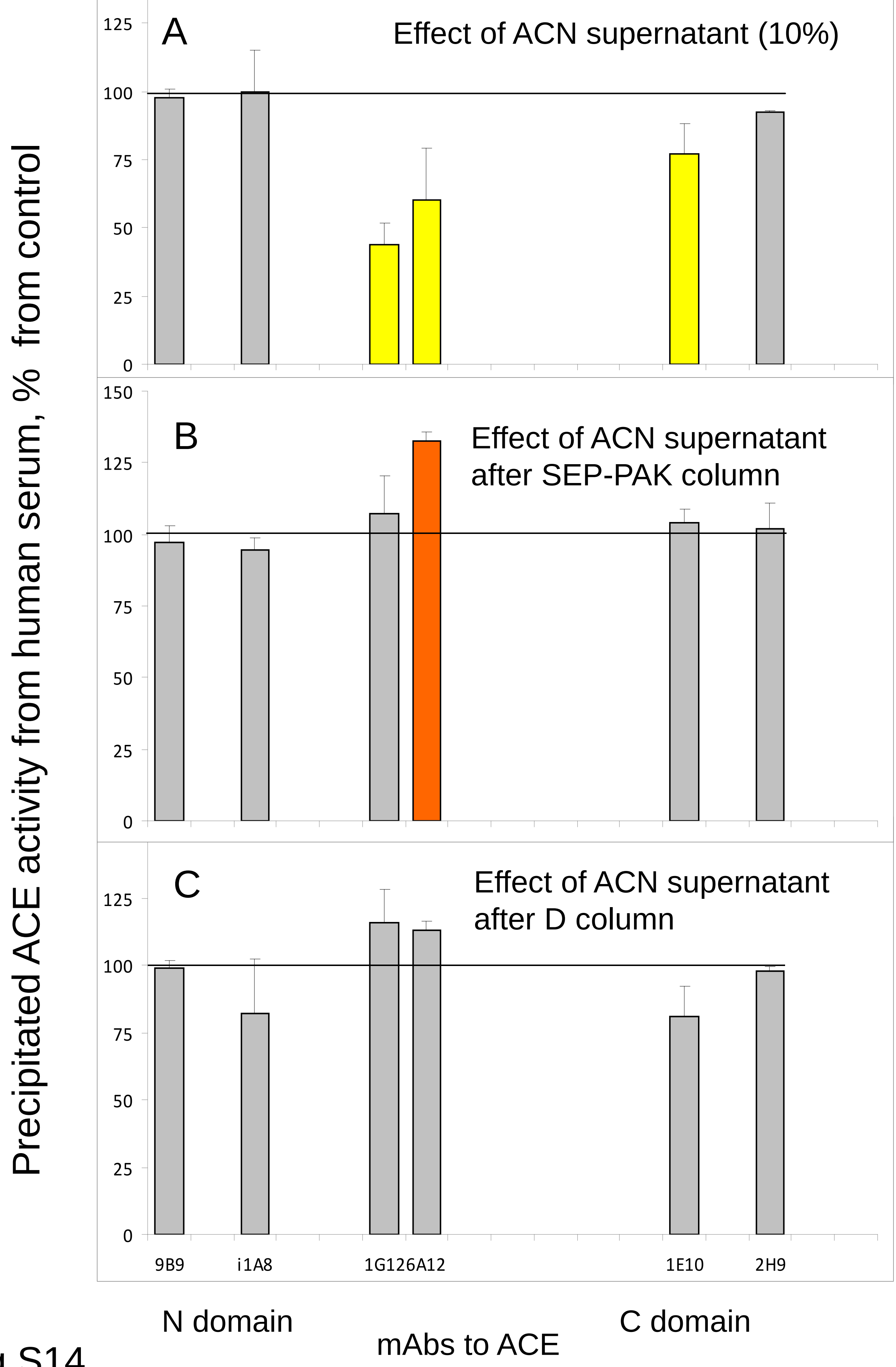
A
Effect of ACN supernatant (10%)
B
Effect of ACN supernatant after SEP-PAK column
C
Effect of ACN supernatant after D column
Precipitated ACE activity from human serum, % from control
0
25
50
75
100
125
150
9B9
i1A8
1G12
6A12
1E10
2H9
N domain
C domain
mAbs to ACE

Fig.S14

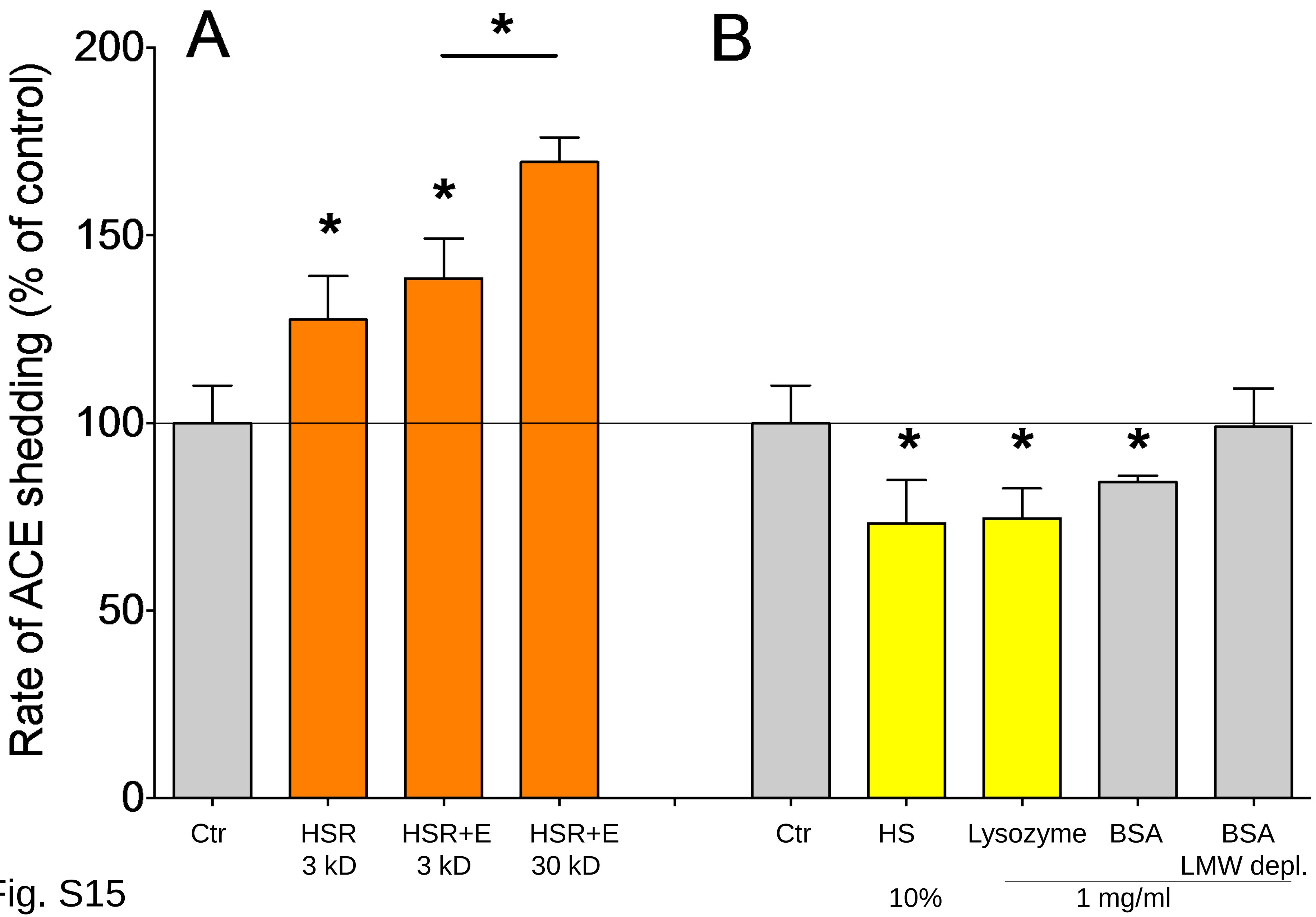
A
B
*
200
150
100
50
0
Rate of ACE shedding (% of control)
*
*
*
*
*
Ctr
HSR
3 kD
HSR+E
3 kD
HSR+E
30 kD
Ctr
HS
Lysozyme
BSA
BSA
LMW depl.
10%
1 mg/ml

Fig. S15

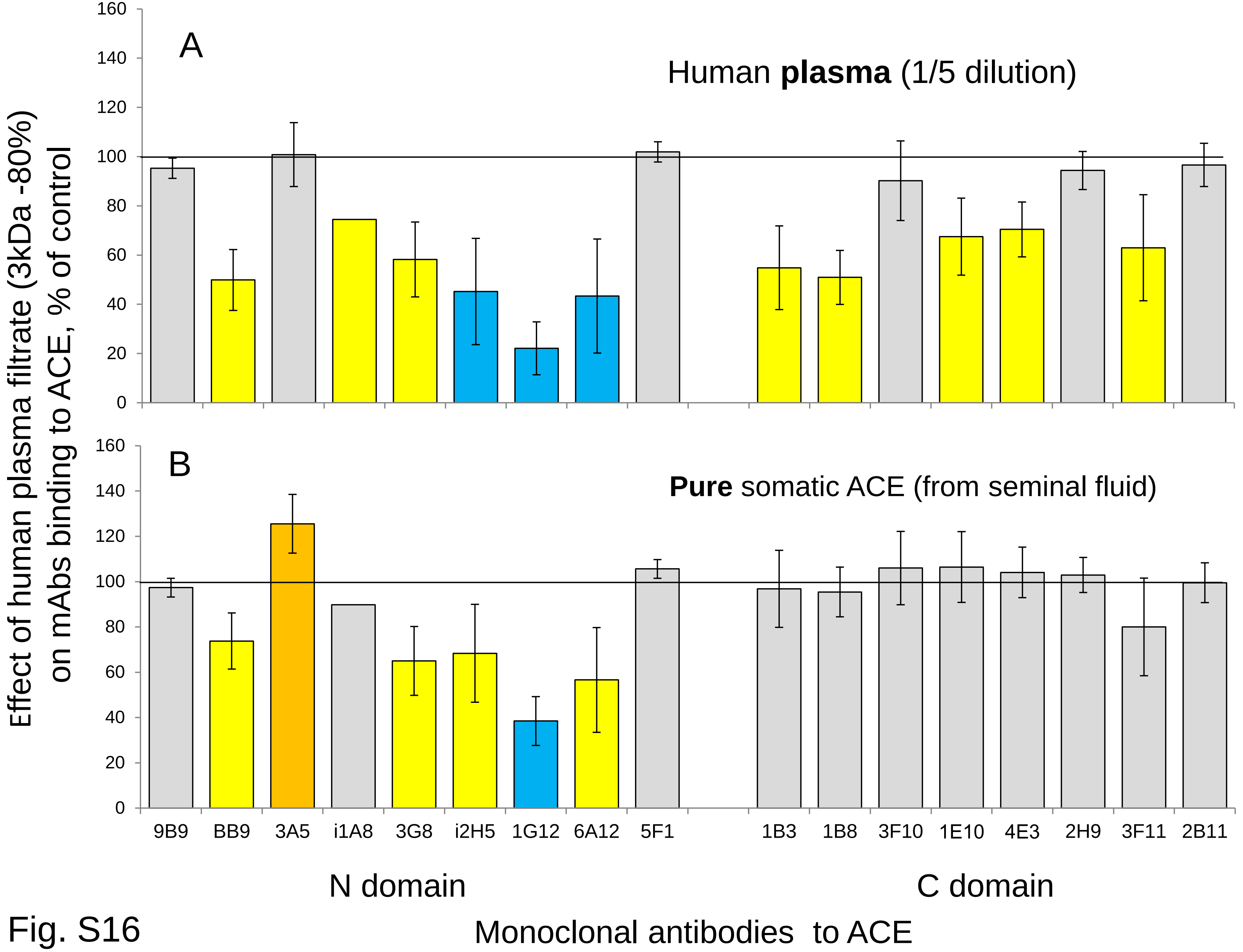
A
Human plasma (1/5 dilution)
B
Pure somatic ACE (from seminal fluid)
Effect of human plasma filtrate (3kDa -80%) on mAbs binding to ACE, % of control
0
20
40
60
80
100
120
140
160
9B9
BB9
3A5
i1A8
3G8
i2H5
1G12
6A12
5F1
1B3
1B8
3F10
1E10
4E3
2H9
3F11
2B11
N domain
C domain
Monoclonal antibodies to ACE

Fig. S16

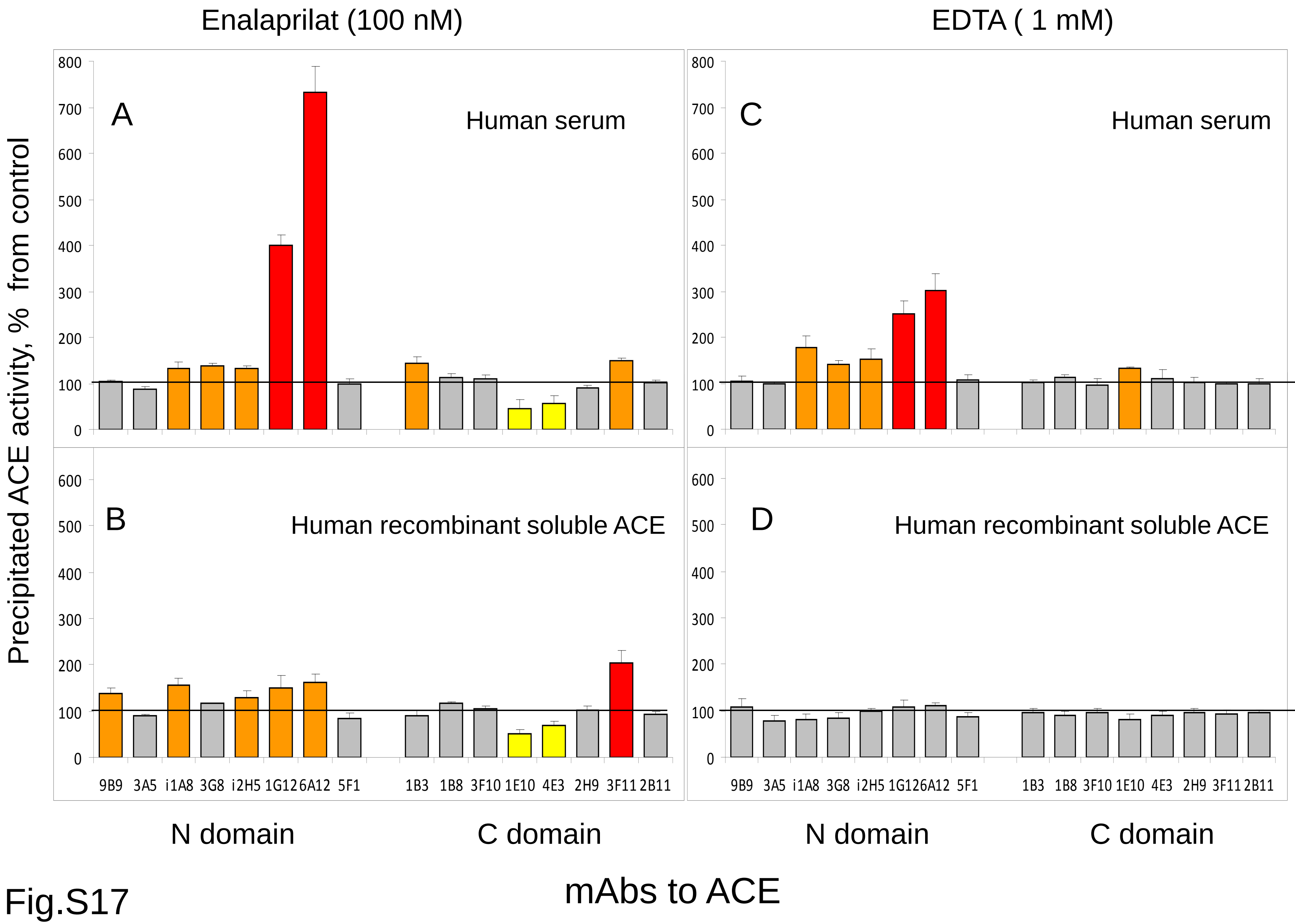

Enalaprilat (100 nM)
EDTA (1 mM)
Precipitated ACE activity, % from control
A
Human serum
C
Human serum
B
Human recombinant soluble ACE
D
Human recombinant soluble ACE
800
700
600
500
400
300
200
100
0
9B9 3A5 i1A8 3G8 i2H5 1G12 6A12 5F1 1B3 1B8 3F10 1E10 4E3 2H9 3F11 2B11
N domain
C domain
mAbs to ACE

Fig.S17

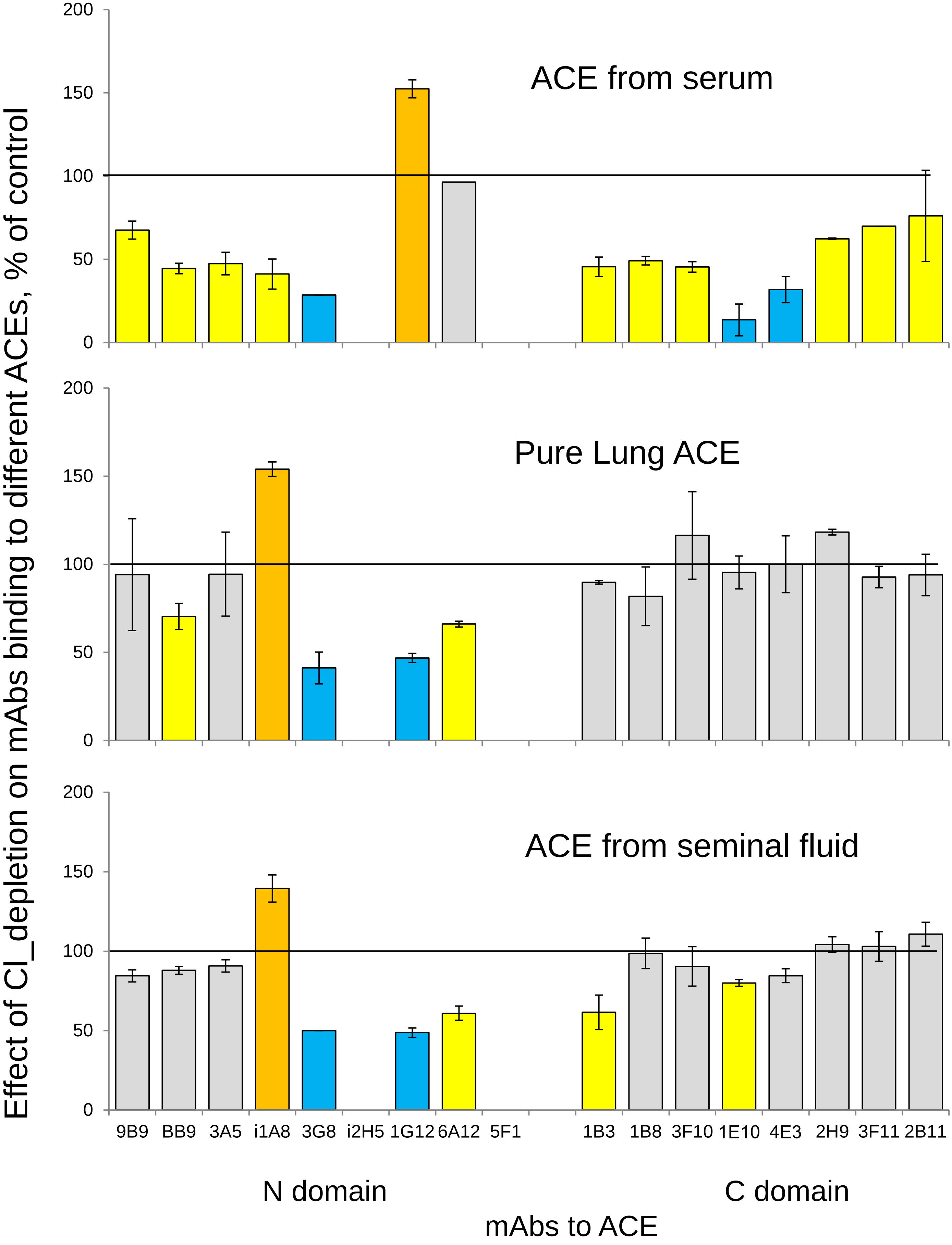
Effect of Cl_depletion on mAbs binding to different ACEs, % of control
ACE from serum
Pure Lung ACE
ACE from seminal fluid
0
50
100
150
200
9B9
BB9
3A5
i1A8
3G8
i2H5
1G12
6A12
5F1
1B3
1B8
3F10
1E10
4E3
2H9
3F11
2B11
N domain
C domain
mAbs to ACE

Fig. S18

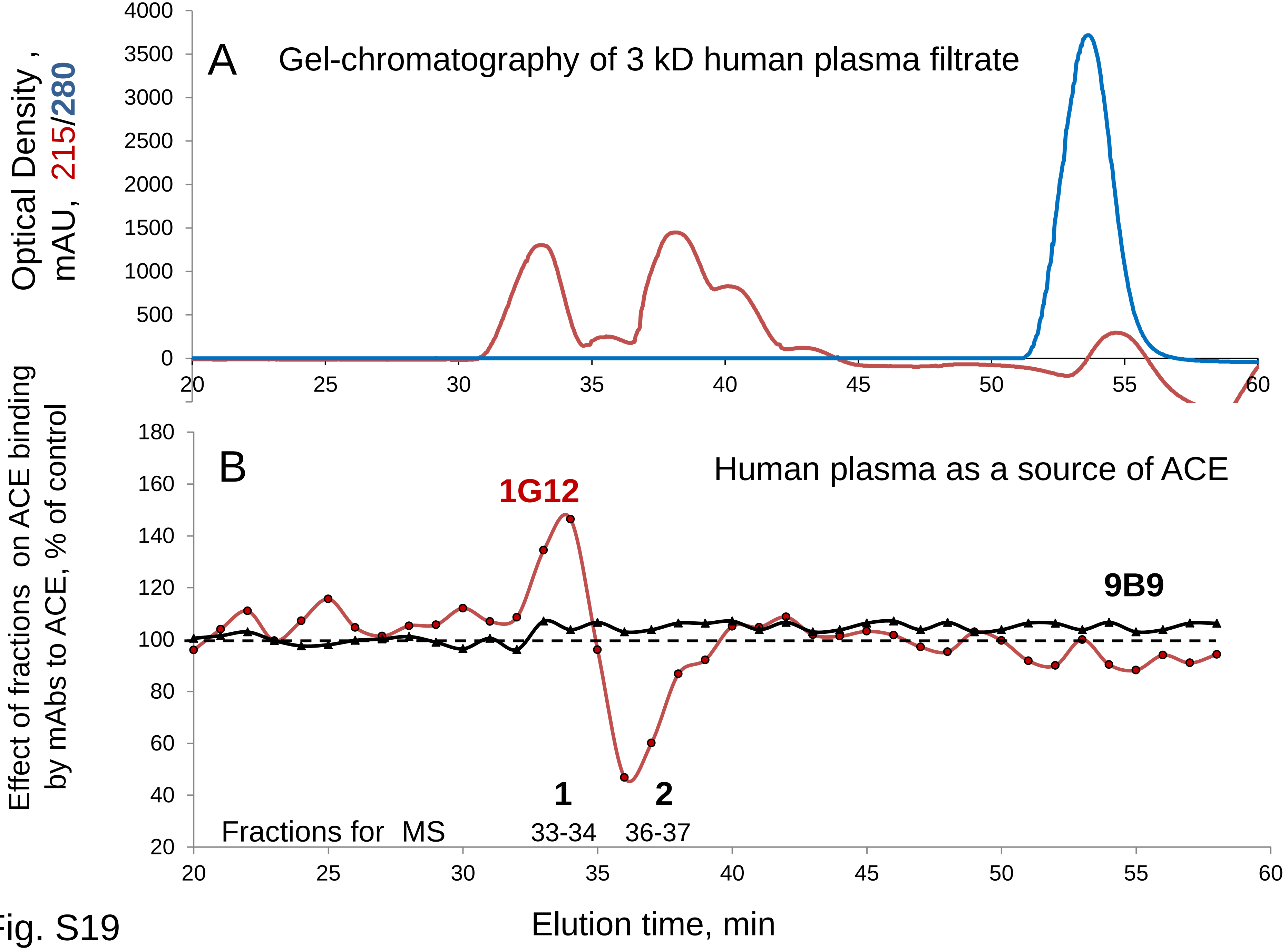

Optical Density , mAU, 215/280
A Gel-chromatography of 3 kD human plasma filtrate
4000
3500
3000
2500
2000
1500
1000
500
0
20 25 30 35 40 45 50 55 60
Effect of fractions on ACE binding by mAbs to ACE, % of control
B Human plasma as a source of ACE
1G12
9B9
180
160
140
120
100
80
60
40
20
1 2
Fractions for MS 33-34 36-37
20 25 30 35 40 45 50 55 60
Elution time, min

Fig. S19
